# Doxorubicin‐Loaded Metal–Organic Framework for Ferroptosis‐Enhanced Chemotherapy Through Sustained Zn Release and Glutathione Peroxidase Downregulation

**DOI:** 10.1002/adhm.202503611

**Published:** 2026-01-04

**Authors:** Xin Ma, Chenghua Deng, Chaoyu Wang, Langston Tilman, Jinhong Li, Wenbin Lin

**Affiliations:** ^1^ Department of Chemistry The University of Chicago Chicago Illinois USA; ^2^ Department of Radiation and Cellular Oncology and Ludwig Center For Metastasis Research The University of Chicago Chicago Illinois USA; ^3^ Department of Chemistry Westlake University Hangzhou Zhejiang China

**Keywords:** chemotherapy, drug delivery, ferroptosis, metal–organic frameworks, mitochondria targeting

## Abstract

As a cornerstone of cancer treatment, chemotherapy is frequently hindered by poor tumor specificity, systemic toxicity, and the emergence of drug resistance. These limitations underscore the need for innovative therapeutic strategies that can circumvent resistance mechanisms and enhance cancer cell cytotoxicity. Herein, we report the development of a structurally robust zinc‐based metal–organic framework (ZnMOF) constructed from 4,4′‐di(pyrazol‐4‐yl)‐1,1′‐biphenyl ligands for simultaneous ferroptosis induction and chemotherapeutic delivery. Compared to the widely used ZIF‐8, the newly developed ZnMOF exhibits superior structural stability under physiological conditions, robust doxorubicin (DOX) loading, and pH‐responsive drug release in acidic tumor microenvironments. In addition to efficient DOX delivery, ZnMOF effectively promotes ferroptosis by elevating intracellular reactive oxygen species, depleting glutathione, and inducing lipid peroxidation. In vivo studies using CT26 and MC38 colon carcinoma models demonstrated potent antitumor efficacy of DOX‐loaded ZnMOF, achieving tumor growth inhibition values of 0.91 and 0.93, respectively. These results position ZnMOF as a promising multifunctional nanoplatform for overcoming chemoresistance and enhancing therapeutic outcomes through ferroptosis‐based combination cancer therapy.

## Graphical abstract

A robust zinc‐based metal–organic framework (ZnMOF) enables dual functions of doxorubicin delivery and sustained Zn^2+^ release to trigger ferroptosis‐enhnaced chemotherapy. DOX@ZnMOF effectively depletes intracellular glutathione, suppresses GPX4, and elevates reactive oxygen species, leading to efficient oxidative DNA damage and apoptosis. In murine colorectal carcinoma models, DOX@ZnMOF achieves potent tumor growth inhibition and significantly prolongs survival, highlighting its potential as a promising nanoplatform for synergistic cancer therapy.

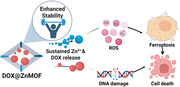

## 1 Introduction

Cancer remains a leading global health burden, accounting for millions of deaths each year despite significant advances in diagnostics and therapeutics [1, 2]. Among current treatments, chemotherapy remains a foundational approach; however, its clinical efficacy is often compromised by poor tumor selectivity, systemic toxicity, and the frequent development of drug resistance [3, 4]. These limitations underscore the urgent need for innovative therapeutic strategies capable of circumventing resistance pathways and enhancing cancer cell cytotoxicity.

An emerging strategy in cancer treatment involves ferroptosis, a regulated form of cell death distinct from apoptosis, necroptosis, and autophagy [5, 6]. Ferroptosis is typically iron‐dependent and driven by the accumulation of lipid peroxides that compromise membrane integrity and induce cell death [7, 8, 9]. While traditionally associated with iron‐mediated Fenton chemistry and the generation of hydroxyl radicals (•OH) from hydrogen peroxide, recent studies revealed that other transition metals, particularly zinc, can also initiate ferroptosis through distinct oxidative pathways [10, 11, 12]. Unlike iron, zinc does not undergo redox cycling and instead induces ferroptosis by disrupting cellular antioxidant defenses. Zn^2+^ has been shown to deplete glutathione (GSH), inhibit glutathione peroxidase 4 (GPX4), and elevate reactive oxygen species (ROS) [10], culminating in lipid peroxidation, a hallmark of ferroptosis. These findings suggest that Zn‐based systems may serve as ferroptosis inducers independent of iron, offering a novel therapeutic avenue for treating drug‐resistant cancers.

Nanoscale metal–organic frameworks (MOFs) have emerged as a promising class of drug carriers due to their large surface areas, high crystallinity, tunable chemical compositions, and tailorable pore environments [13, 14, 15, 16, 17, 18, 19]. These features enable MOFs to achieve high drug‐loading capacities, controlled and stimuli‐responsive release, and enhanced bioavailability, thereby overcoming key limitations of conventional nanocarriers [19, 20, 21, 22, 23, 24, 25]. Among them, ZIF‐8, a zeolitic MOF with the formula {[Zn(2‐methylimidazol‐1‐ide)_2_]}_n_ and sodalite (**sod**) topology, is particularly notable. ZIF‐8 nanoparticles can be readily synthesized by strring an aqueous solution of 2‐methylimidazole and Zn(NO_3_)_2_ at room temperature [26, 27, 28, 29, 30]. Owing to its ease of synthesis, biocompatibility, and pH‐responsive decomposition under acidic tumor microenvironments, ZIF‐8 has been widely used as a MOF‐based nanocarrier, with over 540 publications on its anticancer applications (PubMed search, July 2025) [31, 32, 33, 34]. However, the rapid degradation of ZIF‐8 under physiological conditions has posed challenges for its broader therapeutic utility [35, 36]. In contrast, MOFs constructed from bipyrazole ligands and divalent transition metal cations have demonstrated significantly improved structural stability [37, 38, 39, 40, 41, 42], making them attractive candidates for biomedical applications [43, 44, 45, 46].

**FIGURE 1:**
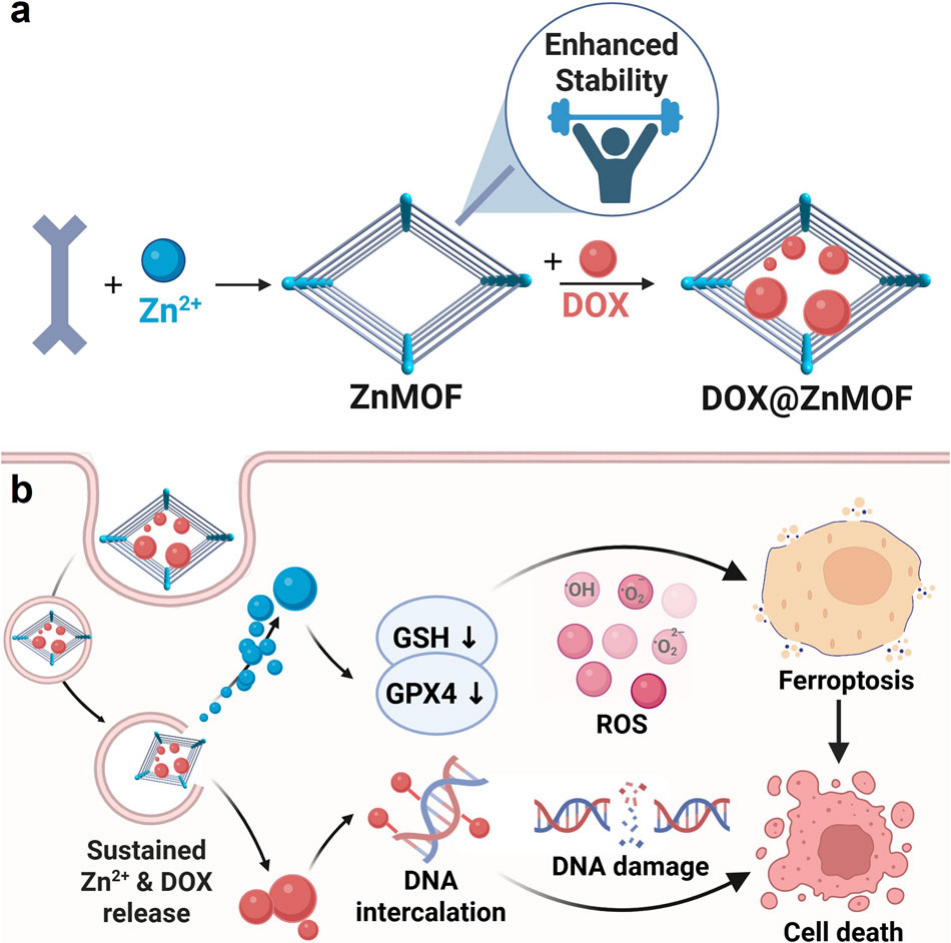
Schematic illustration for (a) the synthesis of ZnMOF and (b) its application as a drug delivery platform for ferroptosis‐enhanced chemotherapy. Created with BioRender.com.

Herein, we report the development of a robust zinc‐based MOF (ZnMOF) constructed from 4,4'‐di(pyrazol‐4‐yl)‐1,1'‐biphenyl (**L**) ligands for applications in drug delivery and cancer therapy. This ZnMOF is specifically engineered for enhanced structural stability under physiological conditions and superior ferroptosis‐inducing capabilities (Figure 1). Compared to ZIF‐8, ZnMOF exhibits markedly improved structural integrity, more robust drug loading, and controlled, pH‐responsive drug release under acidic tumor microenvironments. Doxorubicin (DOX), a widely used chemotherapeutic agent, was incorporated into ZnMOF to evaluate its therapeutic potential. In addition to facilitating mitochondrial targeting to enhance DOX cytotoxicity, ZnMOF effectively promotes ferroptosis by significantly elevating intracellular ROS, depleting glutathione (GSH), and increasing lipid peroxidation, which are hallmark features of ferroptosis‐mediated cell death (Figure 1). In vivo, DOX‐loaded ZnMOF (DOX@ZnMOF) demonstrated potent antitumor efficacy, yielding tumor growth inhibition (TGI) values of 0.91 and 0.93 in CT26 and MC38 colon carcinoma models, respectively.

## 2 Results and Discussion

### 2.1 ZnMOF Synthesis and DOX Loading

**FIGURE 2:**
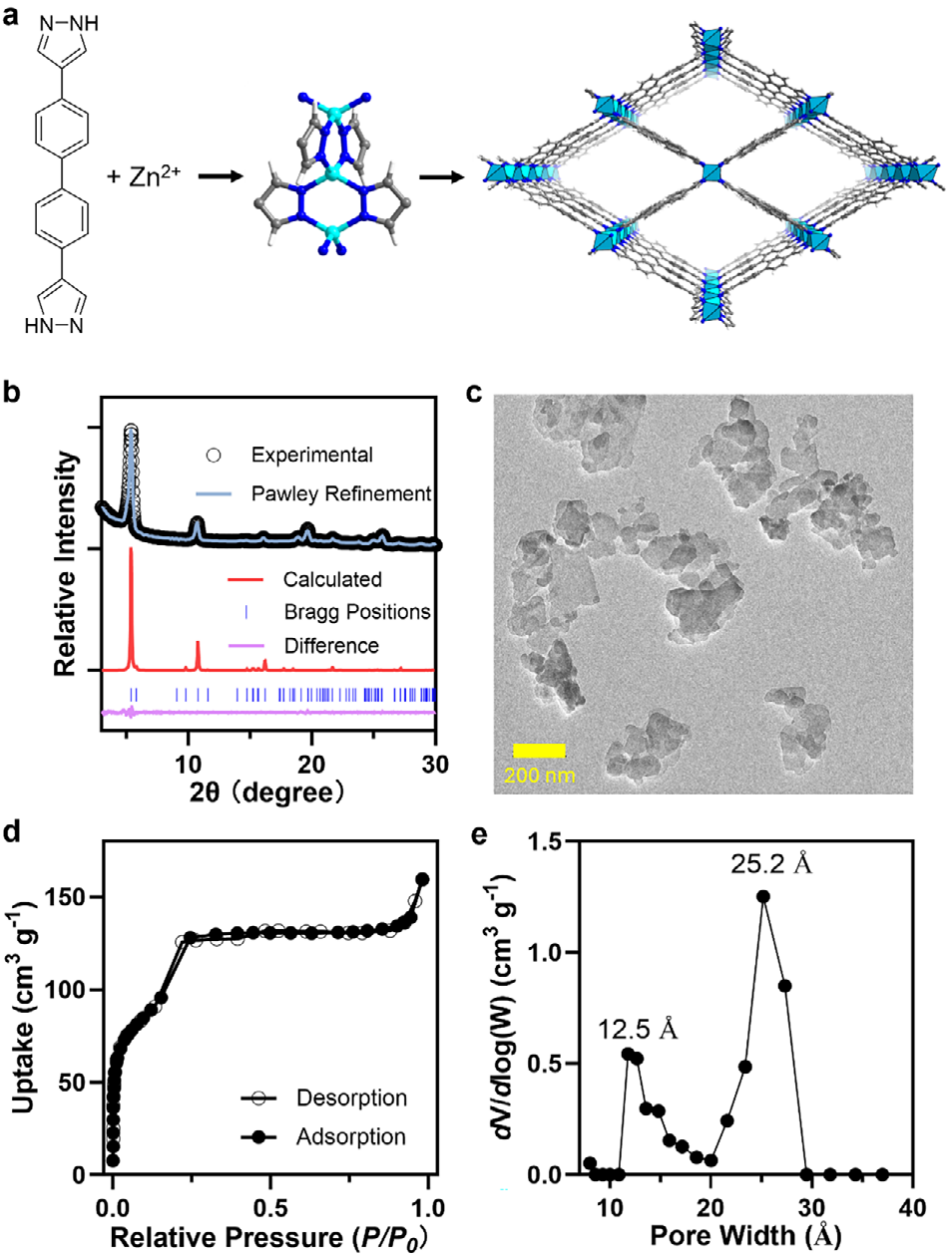
Characterization of ZnMOF. (a) Schematic showing the construction of ZnMOF highlighting its periodic connectivity and porosity. (b) Experimental (blue) and calculated (red) PXRD patterns of ZnMOF. (c) TEM image of ZnMOF. (d) Nitrogen sorption isotherms of ZnMOF at 77 K, (e) Pore size distribution showing dominant pore widths at approximately 12.5 and 25.2 Å.

4,4'‐Di(1*H*‐pyrazol‐4‐yl)‐1,1'‐biphenyl (H_2_ **L**) was synthesized via a Suzuki coupling reaction between 4,4'‐dibromobiphenyl and 1‐(tetrahydro‐2H‐pyran‐2‐yl)‐4‐(4,4,5,5‐tetramethyl‐1,3,2‐dioxaborolan‐2‐yl)‐1H‐pyrazole, followed by acid‐cayalyzed hydrolysis, affording the desired ligand in an overall yield of 79% (Figures S1–S3). ZnMOF nanoparticles were obtained through a solvothermal reaction of H_2_ **L** and Zn(NO_3_)_2_.6H_2_O at 80°C under constant stirring (Figure 2a). The crystalline structure of ZnMOF was characterized by powder X‐ray diffraction (PXRD), and the resulting pattern was analzyed and fitted to determine the framework structure (Figure 2b). A structural model was constructed based on the host framwork of Znbdp (bdp is 4,4'‐(1,4‐phenylene)bis(pyrazol‐1‐ide)) [47], with **L** substituting bdp and the unit cell parameters adjusted accordingly. This model was then subjected to geometry optimization, energy minimization, and Pawley refinement using the Forcite and Reflex modules in the Materials Studio software. The experimental PXRD pattern showed excellent agreement with the refined model, with *R_wp_* and *R_p_* values of 2.69% and 2.07%, respectively.

ZnMOF crystallized in the monoclinic *Pc* space rgoup (Table S1). Zn^2+^ cations are coordinated by pyrazolate anions to form rod‐shaped secondary building units (SBUs), which are further connected through **L** ligands into a 3D **cds** (CdSO_4_) network (Figure 2a). Thermogravimetric analysis (TGA) showed that the framework remained stable up to 430°C (Figure S4). Inductively coupled plasma‐mass spectrometry (ICP‐MS) revealed that zinc constituted 8.75 wt.% of the dried ZnMOF. The ligand content was determined to be 91.7 ± 0.8 wt.% by liquid chromatography‐mass spectrometry (LC‐MS) (Figure S5) and a similar value of 92.3 ± 1.6 wt.% by UV–vis absorption spectroscopy (Figure S6). Based on these results, the molecular formula of ZnMOF is proposed as {[Zn(**L**)](H_2_ **L**)_1.5_}_n_. The presence of uncoordinated H_2_ **L** molecules is attributed to their poor solubility, resulting in partial retention within the MOF channels despite exhasutive washing with various solvents.

**FIGURE 3:**
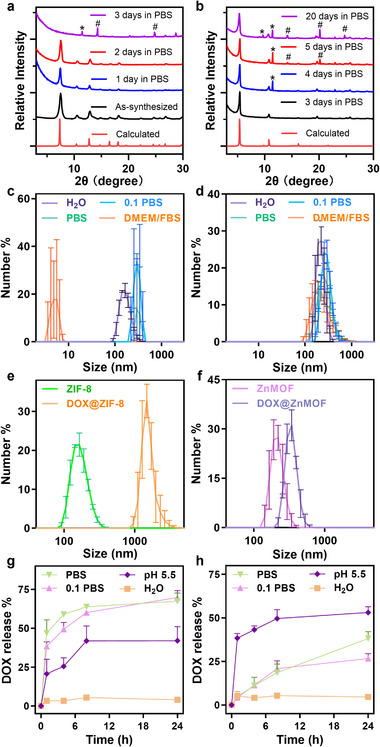
PXRD patterns of (a) ZnMOF and (b) ZIF‐8 after soaking in PBS for 20 and 3 days, respectively. The emerging peaks labelled by ^*^ and # correspond to Na[ZnPO_4_](H_2_O) (ICSD81368) and Na_3_[ZnPO_4_]_3_(H_2_O)_4_ (ICSD63495), respectively. Particle size distributions of (c) ZIF‐8 and (d) ZnMOF dispersed in different media (H_2_O, 0.1 × PBS, PBS, DMEM/FBS). DLS‐measured hydrodynamic size distributions of (e) ZIF‐8 and DOX@ZIF‐8 and (f) ZnMOF and DOX@ZnMOF in water. DOX release profiles from (g) DOX@ZIF‐8 and (h) DOX@ZnMOF under different conditions.

Transmission electron microscopy (TEM) confirmed the uniform nanocrystalline morphology of ZnMOF (Figure 2c). The particles exhibited a nanoplate morphology with lateral dimensions in the 100–200 nm range and thickness below 50 nm, which are favorable for biological interactions and cellular uptake. Dynamic light scattering (DLS) revealed a particle size of 180.8 ± 26.4 nm in water for ZnMOF, which is comparable to that of ZIF‐8 (169.8 ± 4.7 nm, Figure 3c,d).

The porosity of ZnMOF was evaluated using nitrogen sorption isotherms at 77 K (Figure 2d), which displayed a type‐I sorption profile with a nitrogen uptake of 133 cm^3^ g^−1^. The Brunauer–Emmett–Teller (BET) surface area was calculated to be 451.5 m^2^/g. Pore size distribution anaylsis using the density functional theory (DFT) method revealed two dominant pore widths centered at 12.5 and 25 Å (Figure 2e), with a total pore volume of 0.219 cm^3^/g. For comparison, ZIF‐8 was prepared following a reported procedure [48], and its concentration was quantified based on ligand content using UV–vis absorption spectroscopy (Figure S7). ZIF‐8 showed a type‐I adsorption isotherm with a nitrogen uptake of 450 cm^3^ g^−1^ at 77 K and a BET surface area of 1371.9 m^2^/g (Figure S8a). Its PXRD pattern and nitrogen sorption behavior matched well with literature reports (Figures 3a; S8) [28]. Pore size distribution analysis indicated ZIF‐8 contains two dominant pore widths at 10.0 and 13.6 Å (Figure S8), and the total pore volume is 0.818 cm^3^ g^−1^. Although ZnMOF exhibited lower nitrogen uptake and total pore volume compared to ZIF‐8, which is likely due to the presence of uncoordinated H_2_ **L** molecules within the framework, it possesses larger pores, which are advantageous for encapsulating bulky chemotherapeutic agents (such as DOX).

To assess the physiological stability of ZnMOF in comparison to ZIF‐8, we monitored their structural integrity at the same Zn concentration (0.34 mm) by PXRD after incubation in phosphate‐buffered saline (PBS, pH 7.4). The characteristic PXRD peaks of ZIF‐8 diminished significantly within 1 day and were nearly absent after 3 days (Figure 3a), indicating rapid degradation. The PXRD pattern of the residual solid revealed the formation of Na[ZnPO_4_](H_2_O) (ICSD81368) and Na_3_[ZnPO_4_]_3_(H_2_O)_4_ (ICSD63495), confirming the transformation of ZIF‐8 into insoluble zinc phosphate salts (Figure S9). In contrast, ZnMOF retained its crystalline structure with only minor peak attenuation after 20 days of incubation (Figure 3b), highlighting its superior stability under physiological conditions. The newly appeared peaks in the ZnMOF sample also corresponded to Na[ZnPO_4_](H_2_O) and Na_3_[ZnPO_4_]_3_(H_2_O)_4_. The degradation behaviors of ZIF‐8 and ZnMOF were quantitatively evaluated by monitoring ligand release during a seven‐day incubation in PBS (Figure S10). For ZIF‐8, the crystallinity was lost on day 3, when the ligand concentration reached 322 µM (47.4% of the total ligand). The ligand release continued, reaching 597 µm (87.8% of the total ligand) by day 7. ZnMOF exhibited much slower degradation, with only 114 µm (13.4% of total ligand) detected after seven days. The enhanced stability of ZnMOF is likely attributed to the increased hydrophobicity and low aqueous solubility of H_2_ **L** compared to 2‐methylimidazole used in ZIF‐8.

We further investigated the colloidal stability of ZnMOF and ZIF‐8 in biologically relevant media. DLS Analysis showed that ZnMOF retained consistent particle size distributions in H_2_O, PBS, 0.1 × PBS, and DMEM With 10% Fetal Bovine Serum (DMEM/FBS) (Figure 3d). In contrast, ZIF‐8 exhibited increased size and significant aggregation, particularly in DMEM/FBS (Figure 3c), indicating poor colloidal stability under physiological conditions

The DOX loading efficiency in ZnMOF and ZIF‐8 was assessed using UV–vis absorption spectroscopy (Figure S11). ZIF‐8 and ZnMOF showed comparable DOX loadings of 17.4 and 13.0 mol%, respectively, based on the Zn content. Notably, the DOX loading of ZnMOF matches the theoretical value estimated from its pore volume (∼16.8 mol% based on Zn), supporting the successful DOX encapsulation within the framework. DLS analysis revealed a moderate increase in particle size from 180.8 ± 26.4 nm to 324.6 ± 114.6 nm following DOX loading into ZnMOF (Figure 3f). In contrast, DOX loading into ZIF‐8 led to severe aggregation, with particle size increasing from 169.8 ± 4.7 nm to 1573 ± 194.0 nm for DOX@ZIF‐8 (Figure 3e).

The release kinetics of DOX from DOX@ZIF‐8 and DOX@ZnMOF were next evaluated under various conditions. Both systems exhibited accelerated drug release under acidic conditions (pH 5.5), with approximately 49.6% and 41.8% DOX release from DOX@ZIF‐8 and DOX@ZnMOF, respectively, within 8 h, compared to ∼5% release in water (Figure 3g,h). Under physiological conditions (PBS and 0.1 × PBS, pH 7.4), DOX@ZIF‐8 released DOX rapidly, reaching ∼70% release within 8 h. In contrast, DOX@ZnMOF demonstrated significantly slower release, with ∼40% in PBS and ∼25% in 0.1 × PBS after 24 h, highlighting its enhanced structural stability and reduced premature drug leakage. Together, these findings demonstrate the superior structural integrity, colloidal stability, and controlled DOX release profile of ZnMOF compared to ZIF‐8, underscoring its potential in drug delivery and cancer therapy.

### 2.2 Cellular Uptake and Mitochondrial Targeting

To access intracellular trafficking of DOX@ZnMOF, we first evaluated the intrinsic cytotoxicity of ZnMOF by determining Zn concentration‐dependent cell viability in two cell lines. The half‐maximal inhibitory concentration (IC_50_) values of ZnMOF were determined to be 116.2 µm in CT26 cells and 390.3 µm in 4T1 cells (Figure S12), indicating minimal cytotoxic effects at concentrations below 50 um.

**FIGURE 4:**
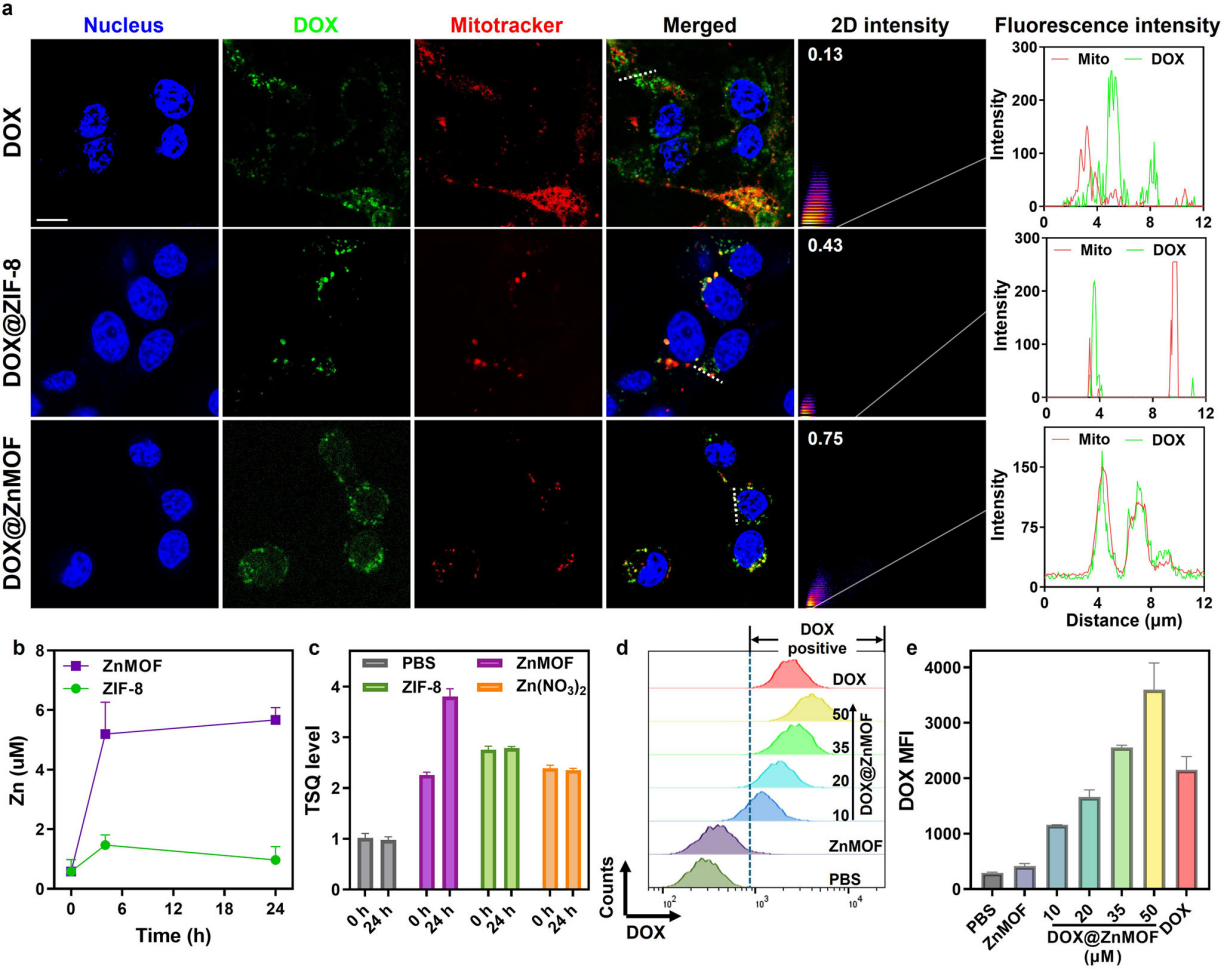
(a) CLSM imaging of CT26 cells treated with free DOX, DOX@ZIF‐8, or DOX@ZnMOF. Channels show nucleus (blue, DAPI), DOX (green), and Mitotracker (red). Merged images and 2D intensity heatmaps reveal differences in intracellular localization. Fluorescence intensity profiles of DOX and Mitotracker signals along the dashed white lines in merged images. Scale bar: 10 µm. (b) Time‐dependent intracellular Zn^2^^+^ concentration measured by ICP‐MS. (c) Quantification of labile Zn^2^^+^ levels in CT26 cells after 4 h incubation with ZnMOF, ZIF‐8, Zn(NO_3_)_2_, or PBS, (*n* = 3), either after immediate TSQ staining (0 h) or after incubation in fresh cell culture medium for 24 h before TSQ staining (24 h). TSQ level of the PBS group was normalized as 1. (d) Flow cytometry histograms of intracellular DOX fluorescence in CT26 cells treated with free DOX, DOX@ZnMOF (10–50 µm), ZnMOF alone, or PBS. (e) Quantification of DOX MFIs from flow cytometry.

Zeta potential analysis showed that ZnMOF and ZIF‐8 exhibited negative surface charges in water. Upon DOX loading, both DOX@ZnMOF and DOX@ZIF‐8 acquired strong positive surface charges under acidic pH conditions (Figure S13). This pH‐responsive surface charge inversion enhances electrostatic interactions with negatively charged cell membranes in the acidic tumor microenvironment, thereby facilitating cellular uptake and mitochondrial targeting. Intracellular Zn^2+^ levels were quantified using ICP‐MS to compare intracellular uptake of ZnMOF vs. ZIF‐8. ZnMOF‐treated CT26 cells exhibited significantly higher Zn^2+^ accumulation than ZIF‐8, with intracellular levels exceeding 6 µm after 24 h compared to < 2 µm for ZIF‐8 (Figure 4b), reflecting the superior cellular internalization of ZnMOF. To investigate Zn^2+^ release dynamics in vitro, CT26 cells were incubated with ZnMOF or ZIF‐8 for 4 h, followed by immediate staining with 6‐methoxy‐(8‐p‐toluenesulfonamido)quinoline (TSQ) or incubation with free cell culture media for 24 h before TSQ staining to assess labile Zn^2+^ concentrations. Cells treated with ZIF‐8 displayed the highest TSQ signal (∼2.76), compared to ZnMOF (∼2.25) and Zn(NO_3_)_2_ (∼2.39), with PBS‐treated cells normalized to 1 (Figures 4c; S14). However, after incubation in fresh cell culture media for 24 h, TSQ intensity in ZnMOF‐treated cells increased substantially to ∼3.80, indicating sustained Zn^2+^ release over time. In contrast, TSQ signals in ZIF‐8 (2.78) and Zn(NO_3_)_2_ (2.35) groups remained almost unchanged, suggesting limited Zn retention or release. These results support ZnMOF's capacity for both efficient cellular uptake and prolonged intracellular Zn^2+^ release.

To further elucidate intracellular trafficking and subcellular localization, confocal laser scanning microscopy (CLSM) imaging was conducted using DOX (green) and MitoTracker (red) staining (Figure 4a). Free DOX showed diffuse cytoplasmic distribution with limited mitochondrial co‐localization (Pearson correlation coefficient (PCC): 0.13). DOX@ZIF‐8 exhibited enhanced cytoplasmic DOX accumulation and moderate mitochondrial overlap (PCC: 0.43), likely reflecting partial targeting due to rapid degradation. In contrast, DOX@ZnMOF displayed strong co‐localization with mitochondria (PCC: 0.75), indicating superior mitochondrial targeting. This improved targeting is attributed to the positive surface charge, smaller particle size, and robust colloidal and structural stability of DOX@ZnMOF, which collectively promote mitochondrial membrane potential‐driven uptake. Enhanced mitochondrial accumulation of DOX is expected to increase mitochondrial ROS production and oxidative damage.

Finally, intracellular DOX accumulation was quantified by flow cytometry. CT26 cells incubated with increasing concentrations of DOX@ZnMOF showed a concentration‐dependent increase in intracellular DOX fluorescence intensity (Figure 4d), with the median fluorescence intensity (MFI) plateauing at higher doses. Notably, DOX@ZnMOF achieved a 1.67‐fold higher MFI compared to free DOX (Figure 4e), confirming its superior cellular uptake and efficient mitochondrial delivery.

### 2.3 Oxidative Stress and Ferroptotic Cell Death

**FIGURE 5:**
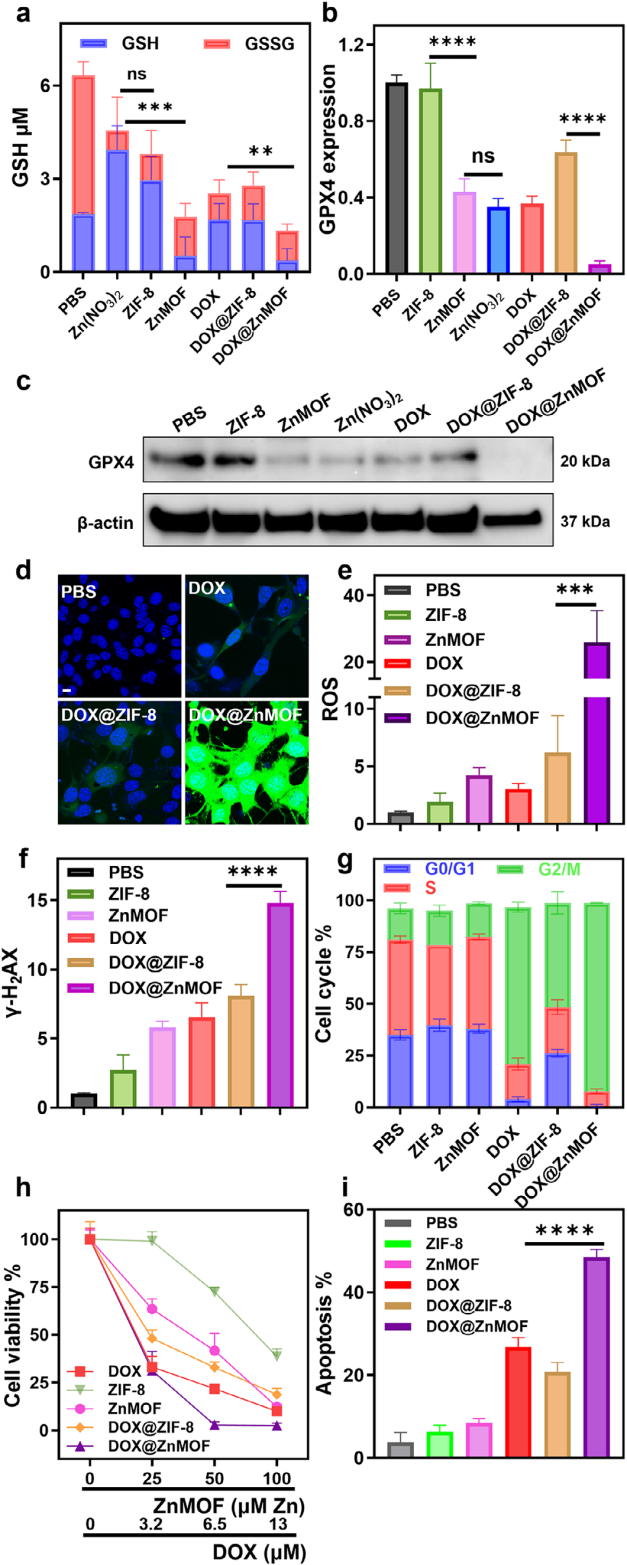
(a) Quantification of intracellular total glutathione (red bars) and GSH (blue bars) after different treatments. *n* = 3. (b) Western blot analysis of GPX4 protein expression after different treatments. The corresponding uncropped images are included in Figure S26. (c) Quantification of relative GPX4 intensities from (b), normalized to β‐actin. (d) CLSM images of CT26 cells comparing intracellular ROS (green) generation in different groups (nuclei stained blue). Scale bar: 10 µm.(e) MFI quantification of total ROS generation after different treatments, (*n* = 3) (f) MFI quantification of γ‐H2AX expression in CT26 cells after different treatments by flow cytometry analysis, (*n* = 3). (g) Cell cycle distribution analysis after different treatments. (*n* = 3) (h) Cell viability assay (MTS) after different treatments for 72 h, *n* = 3. (i) Apoptosis rates of CT26 cells after different treatments, *n* = 3. ^*^ *p* < 0.05, ^**^ *p* < 0.01, ^***^ *p* < 0.001, ^****^ *p* < 0.0001.

GSH, the primary intracellular antioxidant, directly neutralizes ROS and lipid peroxides, thereby preventing ferroptotic cell death [49]. To evaluate oxidative stress, both intracellular GSH and total glutathione (GSH plus glutathione disulfide, GSSG) were quantified. In PBS‐treated CT26 cells, total glutathione was approximately 6.33 µm, with GSH accounting for 1.86 µm (Figure 5a). Treatment with Zn(NO_3_)_2_ resulted in a modest reduction in total glutathione (4.56 µm, ∼28.0% reduction relative to PBS), but unexpectedly increased GSH levels (3.93 µm; ∼111.3% increase), likely reflecting a compensatory antioxidant response triggered by acute Zn^2^^+^ exposure [50]. ZIF‐8 treatment caused a moderate reduction in total glutathione (3.80 µm, ∼40.0% decrease). Strikingly, ZnMOF treatment led to a substantial decrease in both GSH (0.52 µm, ∼72.0% decrease) and total glutathione (1.77 µm; ∼72.0% reduction), highlighting its intrinsic oxidative potential.

Free DOX also significantly depleted total glutathione (2.53 µm, ∼60.0% decrease), consistent with its known mechanisms of DNA intercalation, ROS generation, and oxidative damage [51]. DOX@ZIF‐8 induced a similar level glutathione depletion (2.78 µm, ∼56.1% decrease). In contrast, DOX@ZnMOF triggered profound glutathione depletion, with GSH levels reduced to 0.37 µm (∼80.1% decrease) and total glutathione to 1.33 µm (∼80.0% decrease), suggesting a synergistic effect. The sustained Zn^2+^ release from ZnMOF amplifies oxidative stress by disrupting glutathione biosynthesis and regeneration, accelerating GSH oxidation into GSSG. The persistent ROS burden, enhanced by both Zn^2+^ ions and DOX, likely overwhelms cellular glutathione reductase activity, leading to pronounced depletion of both reduced and oxidized glutathione pools.

To probe ferroptosis induction, we evaluated intracellular lipid peroxidation using a lipid ROS‐specific fluorescent probe, Liperfluo. Flow cytometry analysis showed a ∼1.9‐fold increase in lipid peroxidation following ZnMOF exposure, whereas ZIF‐8 induced a modest ∼1.3‐fold increase. Co‐treatment with the ferroptosis inhibitor Ferrostatin‐1 (Fer‐1) and ZnMOF partially reversed this effect, reducing lipid peroxide levels to ∼1.4‐fold over PBS (Figure S15). These data support that lipid ROS accumulation in ZnMOF‐treated cells is at least partially ferroptosis‐dependent. We then examined the expression of glutathione peroxidase 4 (GPX4) [52], a key enzyme responsible for reducing lipid peroxides. Western blot analysis revealed significant downregulation of GPX4 expression across treatment groups, with densitometric quantification demonstrating reductions of approximately 3.0% (ZIF‐8), 57.0% (ZnMOF), 64.9% (Zn(NO_3_)_2_), 63.3% (DOX), and 36.3% (DOX@ZIF‐8), relative to the PBS control (Figure 5b,c). Notably, DOX@ZnMOF treatment led to a dramatic 94.9% reduction in GPX4 expression, indicating severe impairment of lipid peroxide detoxification and strongly supporting a ferroptotic mechanism.

Interestingly, although Zn(NO_3_)_2_ induced only moderate total glutathione depletion and even elevated GSH, it caused substantial GPX4 downregulation (∼64.9%). This suggests that free Zn^2^^+^ may directly destablize GPX4 or accelerate its degradation independent of glutathione metabolism. In contrast, ZIF‐8 treatment reduced total glutathione but had minimal impact on GPX4 expression, implying that oxidative stress induced by gradual Zn^2+^ release primarily depletes GSH without affecting GPX4 stability. These findings highlight distinct oxidative stress mechanisms triggered by free vs. framework‐bound Zn^2+^. While both converge on ferroptosis, they do so through different molecular targets and temporal dynamics, with DOX@ZnMOF exerting the most potent and synergistic ferroptotic effect through sustained Zn^2+^ release and robust GPX4 suppression.

We next examined intracellular ROS accumulation using the ROS‐sensitive fluorescent probe DCFH‐DA. DOX@ZnMOF and DOX@ZIF‐8 treatments markedly elevated ROS levels compared to free DOX (Figures 5d,e; S16). Treatment with ZnMOF or ZIF‐8 alone led to 4.24 and 1.89‐fold increases in DCFH MFI relative to the PBS control, respectively. The addition of DOX further amplified ROS generation by Zn‐based MOFs; DOX@ZnMOF and DOX@ZIF‐8 induced 8.55 and 2.05‐fold increases in DCFH MFI, respectively, compared to free DOX. This substantial ROS elevation in the DOX@ZnMOF group underscores the potent oxidative environment generated by sustained Zn^2+^ release, which overwhelms cellular antioxidant defenses and drives ferroptotic cell death.

### 2.4 In Vitro Anticancer Effect

We assessed the genotoxic impact of DOX@ZnMOF by analyzing DNA damage using γ‐H2AX immunofluorescence, a sensitive biomarker of DNA double‐strand breaks [53]. DOX@ZnMOF treatment significantly increased γ‐H2AX signal, showing a 2.23‐fold increase compared to free DOX (Figures 5f; S17), whereas DOX@ZIF‐8 only induced a moderate 1.23‐fold enhancement. This pronounced DNA damage correlates closely with the elevated ROS levels observed in DOX@ZnMOF‐treated cells, reinforcing the role of oxidative stress in mediating ferroptosis. Additionally, cell cycle analysis revealed that DOX@ZnMOF induced significant G2/M phase arrest, with the G2/M population increasing to 90.9%, from 50.1% in the DOX@ZIF‐8 group (Figures 5g; S18). This marked cell cycle disruption indicates activation of DNA damage response pathways and supports ferroptosis‐associated cell death induced by DOX@ZnMOF.

To evaluate therapeutic efficacy, we conducted MTS assays at 24 and 72 h post treatments. At 24 h, DOX@ZnMOF exhibited cytotoxicity comparable to free DOX, both inducing dose‐dependent reductions in cell viability (Figure S19). At a dose of 6.5 µm DOX (corresponding to 50 µm ZnMOF), DOX@ZnMOF reduced cell viability to 42.2%, similar to the 32.2% viability observed with free DOX. In contrast, DOX@ZIF‐8 resulted in higher residual viability (59.5%), likely due to suboptimal cellular uptake as a result of particle aggregation.

By 72 h, DOX@ZnMOF demonstrated the highest cytotoxicity among all treatments (Figure 5h). At the highest dose (100 µM ZnMOF / 13 µM DOX), DOX@ZnMOF reduced cell viability to 2.5%, significantly outperforming DOX@ZIF‐8 (18.7%) and free DOX (9.9%). At intermediate doses (50 µM ZnMOF / 6.5 µM DOX), DOX@ZnMOF maintained potent cytotoxicity, reducing viability to 2.9%, compared to 32.9% for DOX@ZIF‐8. These findings underscore the superior long‐term therapeutic efficacy of DOX@ZnMOF, likely due to sustained Zn2+ and DOX release as well as effective ferroptosis induction. In contrast, the limited cytotoxicity of DOX@ZIF‐8 reflects its poor cellular uptake and drug retention. Consistent with these findings, DOX@ZnMOF also induced the highest level of apoptosis (Figures 5i; S20), with 48.47% apoptotic cells. Apoptosis rates were signifcantly lower in the DOX@ZIF‐8 (20.83%), free DOX (26.82%), ZnMOF (8.42%), and ZIF‐8 (6.23%) groups.

### 2.5 In Vivo Antitumor Efficacy

To validate the therapeutic potential of DOX@ZnMOF in vivo, we employed two murine colorectal cancer models: CT26‐bearing BALB/c mice and MC38‐bearing C57BL/6 mice. Once tumors reached ∼100 mm^3^ (day 7 post‐inoculation), mice were intratumorally injected with PBS, ZIF‐8, ZnMOF, DOX, DOX@ZIF‐8, or DOX@ZnMOF at a Zn dose of 3 µmol and/or DOX dose of 0.39 µmol on days 7 and 8 (Figure S21). The tumor growth inhibition index (TGI) was defined as:

$$\mathrm{TGI}=\left[1-\left(\mathrm{Te}-\mathrm{Ts}\right)/\left(\mathrm{Ce}-\mathrm{Cs}\right)\right]\times 100\%$$

where 𝑇𝑒, 𝑇𝑠, 𝐶𝑒, and 𝐶𝑠 represent average tumor volumes of treated mice at the endpoint, treated mice at the starting‐point, control mice at the endpoint and control mice at the starting‐point, respectively.

**FIGURE 6:**
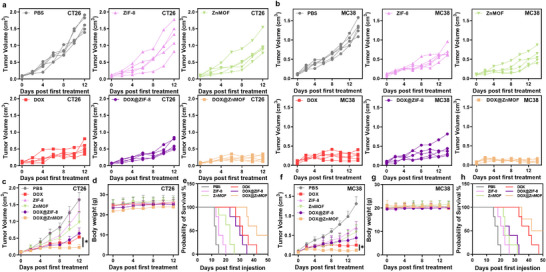
(a, b) Individual tumor growth curves in (a) CT26‐bearing, and (b) MC‐38‐bearing mice across all groups, (*n* = 5). (c–e) (c) Tumor growth curves, (d) body weight, and (e) survival curves of subcutaneous CT26 tumor‐bearing BALB/c mice after different treatments. (*n* = 5). (f–h) (f) Tumor growth curves, (g) body weight, and (h) survival curves, of subcutaneous MC38 tumor‐bearing C57 mice after different treatments. (*n* = 5).^*^ *p* < 0.05, ^**^ *p* < 0.01, ^***^ *p* < 0.001, ^****^ *p* < 0.0001.

In the CT26 model, DOX@ZnMOF elicited the most pronounced tumor suppression, with a tumor growth inhibition (TGI) value of 0.91, which is substantially higher than that of free DOX (TGI = 0.70) and significantly outperforming both DOX@ZIF‐8 (TGI = 0.48) and ZnMOF alone (TGI = 0.38). ZIF‐8 exhibited the weakest therapeutic response (TGI = 0.24). These results are consistent with the rapid degradation and poor drug retention of ZIF‐8 in vivo (Figure 6a,c; Tables S2 and S4). Body weights remained stable across all groups (Figure 6d), indicating minimal systemic toxicity. Survival analysis corroborated these findings: DOX@ZnMOF extended the median survival time to 42 days, significantly longer than free DOX (31 days) and PBS (14 days) (Figure 6e), affirming the dual therapeutic benefits of DOX@ZnMOF via ferroptosis induction and chemotherapeutic activity.

A similar therapeutic trend was observed in the MC38 model, where DOX@ZnMOF again achieved the highest TGI of 0.93, outperforming DOX@ZIF‐8 (TGI = 0.56) and free DOX (TGI = 0.76) (Figure 6f; Tables S3 and S5). DOX@ZnMOF treatment also prolonged median survival beyond 50 days, with 60% of treated mice remaining alive at the study endpoint, compared to 41 days for free DOX and 17 days for PBS (Figure 6h). All groups maintained stable body weight throughout the study (Figure 6g), supporting the good biocompatibility of the ZnMOF platform.

**FIGURE 7:**
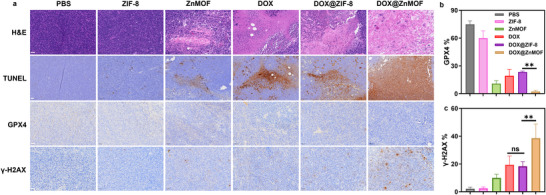
(a) H&E, TUNEL, and immunohistochemistry staining of GPX4 and γ‐H2AX of excised CT26 tumor sections in different treatment groups (Nuclei stained by hematoxylin in blue; TUNEL positive by 3,3’‐Diaminobenzidine (DAB) in red; scale bar = 100 µm) Quantification of (b) GPX4 and (c) γ‐H2AX positive cells across treatment groups, *n* = 5; ^*^ *p* < 0.05, ^**^ *p* < 0.01, ^***^ *p* < 0.001, ^****^ *p* < 0.0001.

To investigate the underlying mechanism of antitumor efficacy, histological and immunohistochemical (IHC) analyses were performed on CT26 tumors. Hematoxylin and eosin (H&E) staining revealed extensive tumor necrosis in the DOX@ZnMOF group, in contrast to the preserved viable tumor architecture seen in PBS‐ and ZIF‐8‐treated tissues (Figure 7a). IHC staining for GPX4 demonstrated most significant downregulation in the DOX@ZnMOF group, with signal intensity approximately 32.5‐fold lower than PBS and ∼10.2‐fold lower than DOX@ZIF‐8 (Figure 7b), confirming robust ferroptosis induction. Additionally, γ‐H2AX staining demonstrated markedly increased DNA double‐strand breaks in the DOX@ZnMOF group, with 2.1– and 2.0–fold higher signals than the DOX@ZIF‐8 and DOX groups, respectively (Figure 7c). Terminal deoxynucleotidyl transferase dUTP nick end labeling (TUNEL) staining revealed the highest apoptotic index in DOX@ZnMOF‐treated tumors (∼82.5% TUNEL‐positive cells), significantly surpassing DOX (∼37.4%) and DOX@ZIF‐8 (∼44.2%) (Figure S22). These results indicate that DOX@ZnMOF induces cell death through a ferroptosis‐ROS axis that synergizes with DOX‐mediated DNA damage.

To assess systemic toxicity, H&E staining was performed on major organs including heart, lungs, liver, spleen, and kidneys from CT26 tumor‐bearing mice (Figure S23). No histopathological abnormalities, inflammatory infiltration, or tissue degeneration were observed across all treatment groups, supporting the excellent biocompatibility and safety profile of DOX@ZnMOF. To further evaluate biosafety, we conducted serum biochemistry and hemolysis assays. The serum levels of aspartate aminotransferase (AST), alanine aminotransferase (ALT), and blood urea nitrogen (BUN) remained within the normal ranges across all treatment groups, indicating no hepatic or renal toxicity following administration (Figure S24). In the hemolysis assay, ZnMOF alone showed negligible hemolysis (< 5%) at up to 3 mm Zn, a dose level that is signficantly higher than the expected blood concentration of ZnMOF at therapeutically relevant doses (Figure S25).

Together, these findings demonstrate that DOX@ZnMOF exerts potent ferroptosis‐enhanced antitumor efficacy in vivo, significantly suppresses tumor progression, prolongs survival, and exhibits minimal systemic toxicity. By combining sustained Zn^2+^ release with chemotherapeutic action, DOX@ZnMOF represents a promising therapeutic strategy for the treatment of colorectal cancer.

## 3 Conclusion

In this work, we developed a novel Zn‐based MOF (ZnMOF) for enhanced DOX delivery and ferroptosis‐mediated cancer therapy. Compared to ZIF‐8, ZnMOF demonstrated superior structural stability and slower degradation kinetics, enabling sustained Zn^2+^ release to drive ferroptosis. Mechanistically, ZnMOF treatment led to significant intracellular glutathione depletion, GPX4 downregulation, elevated ROS accumulation, and pronounced DNA damage and apoptosis when combined with DOX. In vivo, DOX@ZnMOF achieved potent tumor suppression, with TGI values of 0.91 and 0.93 in CT26 and MC38 colorectal cancer models, respectively, while significally extending median survival without detectable systemic toxicity. Histological and immunohistochemical analyses confirmed ferroptosis‐associated lipid peroxidation and enhanced apoptosis in tumors. These findings establish ZnMOF as a multifunctional nanoplatform that integrates chemotherapeutic delivery with ferroptosis induction, offering a promising strategy to overcome treatment resistance and improve therapeutic outcomes in solid tumors.

## Conflicts of Interest

The authors declare no conflicts of interest.

## Supporting information




**Supporting File**: adhm70700‐sup‐0001‐SuppMat.pdf.

## Data Availability

The data that support the findings of this study are available from the corresponding author upon reasonable request.
