# Supplementary material for: Doxorubicin‐Loaded Metal–Organic Framework for Ferroptosis‐Enhanced Chemotherapy Through Sustained Zn Release and Glutathione Peroxidase Downregulation
Source: Adv Healthc Mater. 2026 Jan 4;15(12):e03611. doi: 10.1002/adhm.202503611 (PMC13015774; doi:10.1002/adhm.202503611)
Supplement: Supplementary file 1 — Supporting File: adhm70700‐sup‐0001‐SuppMat.pdf. [file ADHM-15-0-s001.pdf]

Supporting Information

Doxorubicin-Loaded Metal-Organic Framework for Ferroptosis-Enhanced Chemotherapy through Sustained Zn Release and Glutathione Peroxidase Downregulation

Xin Ma,† [a] Chenghua Deng,† [a] Chaoyu Wang,[a,b] Langston Tilman,[a] Jinhong Li,[a] Wenbin Lin*[a,b,c]

[a]Department of Chemistry, The University of Chicago, Chicago, Illinois 60637, United States

[b]Department of Radiation and Cellular Oncology and Ludwig Center for Metastasis Research, The University of Chicago, Chicago, Illinois 60637, United States

Email: wenbinlin@uchicago.edu

[c]Department of Chemistry, Westlake University, 600 Dunyu Road, Hangzhou 310030, Zhejiang, China

E-mail: linwenbin@westlake.edu.cn

KEYWORDS: Metal-organic frameworks • drug delivery • Ferroptosis • Mitochondria targeting • Chemotherapy

Table of Contents

S1 Experimental Section........ 2

S1.1 Materials and Methods........ 2

S1.2. Synthetic Procedures for MOFs........ 3

S1.3. DOX Loading and Release Profiles........ 4

S1.4. In Vitro Studies........ 5

S1.5 In Vivo Study........ 7

S1.6 Statistical Analysis........ 8

S1.7 Ethical Statement........ 8

S2 Supporting Figures and Tables........ 10

References........ 27

S1 Experimental Section

S1.1 Materials and Methods

All starting materials were commercially available and used without further purification unless otherwise noted. Transmission electron microscopy (TEM) was carried out on a TECNAI Spirit and a TECNAI F30 HRTEM. Powder X-ray diffraction (PXRD) data were collected on a Rigaku miniFlex (6th generation) benchtop X-ray diffractometer equipped with a HyPix-400 detector and a Cu Kα X-ray source (1.5406 Å) operating at 40 kV and 15 mA. Powder samples were activated under high vacuum using a Micromeritics Smart VacPrep system at 80 °C overnight prior to gas sorption experiments. Nitrogen sorption isotherms at 77 K were measured with a Micromeritics 3Flex adsorption analyzer, and the temperature was controlled by a 3.2 L dewar of liquid nitrogen. Thermogravimetric analyses (TGA) were performed under air using a TGA-50 SHIMADZU thermogravimetric analyzer. The sample was loaded into an alumina crucible and heated at 10 $°C \cdot min^{-1}$ from room temperature to 800 °C. The concentrations of zinc cations were detect by an Agilent 7700x ICP-MS and analyzed using an ICP-MS Mass Hunter version 4.6 C01.06. The mass spectrum information and concentration of ligand were determined by an Agilent 6540 Q-Tof LCMS system installed with AJS-ESI ion source and 5 μm Agilent C18 column. UV-vis spectra were collected using a Shimadzu UV-2600 UV-Vis spectrophotometer. Dynamic light scattering (DLS) and ζ potential measurements were performed on a Malvern Zetasizer Nano ZS instrument. NMR spectra were recorded on a Bruker Avance III HD nanobay 400 MHz NMR spectrometer. The proton chemical shifts for DMSO-d_6 and Chloroform-d ($CDCl_3$) were fixed at 2.50 and 7.60 ppm, respectively. The ^{13}C chemical shift for CDCl3 was fixed at 77.16 ppm. Flow cytometry data were collected on an LSR-Fortessa 4-15 (BD Biosciences, USA) and analyzed with FlowJo software (Tree Star, USA). Confocal laser scanning microscopy (CLSM) images were collected on a Leica Stellaris 8 laser scanning confocal microscope at the University of Chicago Integrated Light Microscopy Facility and analyzed with ImageJ software (NIH, USA). Histological slides were scanned on a CRi Pannoramic SCAN 40x whole slide scanner by Integrated Light Microscopy Core in the University of Chicago and analyzed with the QuPath-0.2.3 software.[1] The absorbance and fluorescence signals from well plates were read with a BioTek Synergy HTX microplate reader.

Sodium chloride (NaCl, 99%), magnesium sulphate anhydrous ($MgSO_4$, 98%), and potassium carbonate (K_2CO_3, 99%) were purchased from Oakwood Chemical. Ethanol (≥ 99%) was purchased from Decon Laboratories. Zinc nitrate hexahydrate ($Zn(NO_3)_2 \cdot 6H_2O$, 98%), dichloromethane (CH_2Cl_2, 99.5%), dimethyl sulfoxide (DMSO, Certified ACS), tetrahydrofuran (THF, Certified ACS), hexane (98.5%) and ethyl acetate (EA, 99.5%) were purchased from Fisher Scientific. 2-Methylimidazole (99%), tetrakis(triphenylphosphine)palladium ($Pd(PPh_3)_4$, 99%) and hydrochloric acid (HCl, 37%) were purchased from Sigma-Aldrich. Silica 60M (0.04 – 0.063 mm) and pre-coated TLC-plates SIL G-25 were purchased from MACHEREY-NAGEL. $CDCl_3$ and dimethyl sulfoxide-d_6 (DMSO-d_6) were purchased from Cambridge Isotope Laboratories. 4,4'-Dibromobiphenyl (97%) and 1-(tetrahydro-2H-pyran-2-yl)-4-(4,4,5,5-tetramethyl-1,3,2-dioxaborolan-2-yl)-1H-pyrazole (98%) were purchased from Combi-blocks. Trifluoroacetic acid (TFA, > 99.0%) and *N*,*N*-diethylformamide (DEF, > 99%) were purchased from TCI. PBS was purchased from ThermoFisher. Trypsin-EDTA solution was purchased from

the American Type Culture Collection (ATCC, Rockville, MD). 3-(4,5-Dimethylthiazol-2-yl)-5-(3-carboxymethoxyphenyl)-2-(4-sulfo-phenyl)2H-tetrazolium (MTS) was purchased from Promega (USA).

Murine colorectal carcinoma CT26 and MC38 cells were purchased from ATCC. MC38 or CT26 cells were cultured in DMEM (Gibco, USA). or RPMI-1640 (Corning, USA) (Gibco, USA). DMEM and RPMI-1640 media was supported with 10% fetal bovine serum (VWR, USA), 100 U/ml penicillin G sodium and 100 µg/ml streptomycin sulphate. The cells were kept in a humidified atmosphere containing 5% CO_2 at 37°C.

C57 and BALB/c breeders were obtained from Charles River Laboratories (USA) and bred in house at the animal facility at the University of Chicago. C57 and BALB/c mice with an age of 6-8 weeks were used for in vivo experiments. The study protocol was reviewed and approved by the Institutional Animal Care and Use Committee (IACUC) at the University of Chicago (PHS Assurance #D16-00322 (A3523-01)). The Human Tissue Resource Center at the University of Chicago provided the histology related services for this study.

The original model for ZnMOF was built based on the single crystal X-ray structure of Znbdp (bdp is 4,4'-(1,4-phenylene)bis(pyrazol-1-ide), CCDC number: 742072),[2] with the bdp ligand replaced by **L** and the unit cell parameters adjusted accordingly. The model was then subjected to energy minimization and geometry optimization using universal forcefield within the Forcite module of Materials Studio software. Then, Pawley refinements were performed using the Reflex module of Materials Studio software to optimize the structure. The *Rwp* and *Rp* values were 2.69% and 2.07%, respectively.

For dynamic light scattering size and ζ potential measurements, ZnMOF or ZIF-8 samples were dispersed in deionized water at a Zn concentration of 20 µM. 1 mL of the sample was added to a DTS1060 cell and measured on a Malvern Zetasizer Nano ZS instrument.

S1.2. Synthetic Procedures for MOFs

S1.2.1 Synthesis of 4,4'-bis(1-(tetrahydro-2H-pyran-2-yl)-1*H*-pyrazol-4-yl)-1,1'-biphenyl (L-THP). **L**-THP was synthesized through a Suzuki coupling reaction between 4,4'-dibromobiphenyl (1.2 g, 3.85 mmol) and 1-(tetrahydro-2*H*-pyran-2-yl)-4-(4,4,5,5-tetramethyl-1,3,2-dioxaborolan-2-yl)-1*H*-pyrazole (2.57g, 9.24 mmol, 2.4 equiv.) in 100 mL THF/H_2O = 4:1 (v/v) with K_2CO_3 (5 g, 36.18 mmol, 9.4 equiv.) in a 200 mL Schlenk bottle. The mixture in the Schlenk bottle was degassed by three freeze-thaw cycles under vacuum at the liquid nitrogen temperature. Then, $Pd(PPh_3)_4$ (180 mg, 0.16 mmol, 0.04 equiv.) was added to the solution under a nitrogen flow. The solution was refluxed at 80 °C and under a nitrogen flow for 1 day. After cooling to room temperature, the mixture was extracted with ethyl acetate (EA) three times and then washed with deionized water and saturated NaCl solution. The organic phase was dried with anhydrous $MgSO_4$, and the solvent was removed by rotary evaporation. The crude product was purified by column chromatography using silica gel and EA/hexanes as an eluent. The EA/hexanes (v/v) ratio gradually increased from 1:10 to 1:1. The light-yellow powder of Me_4**L**-$(Et)_2$ (1.47 g, 3.23 mmol, ~83.9% yield) was obtained after removal of the solvents with a

rotary evaporator. ^{1}H NMR (400 MHz, $CDCl_3$): δ = 7.90 (s, 2 H), 7.87 (s, 2 H), 7.63-7.61 (d, J = 8.0 Hz, 4 H), 7.57-7.55 (d, J = 8.0 Hz, 4 H), 5.44-5.42 (d, J = 8.0 Hz, 2 H), 4.12-4.09 (d, J = 12.0, 2 H), 3.77-3.72 (t, J = 8.0, 2 H), 2.20-2.04 (m, J = 8.0 Hz, 6 H), 1.77-1.58 (m, J = 8.0 Hz, 6 H). ^{13}C NMR (101 MHz, $CDCl_3$): δ = 138.94, 137.34, 131.62, 127.38, 126.16, 124.60, 123.22, 87.96, 67.97, 30.70, 25.12, 22.54. MS (ESI-TOF): calculated m/z for $[M + H]^+$ ($C_{28}H_{31}N_4O_2$), 455.5820; observed, 455.2453.

S1.2.2 Synthesis of 4,4'-di(1*H*-pyrazol-4-yl)-1,1'-biphenyl (H_2L). 1.4 g **L**-THP (3.08 mmol) was mixed with 20 mL ethanol, 20 mL THF and 4 mL HCl in a 100 mL round-bottom flask, and the mixture was refluxed at 80 °C overnight. After cooling to room temperature, saturated Na_2CO_3 aqueous solution was adding to the mixture till pH = 4. The solid was collected by filtration. The crude product was sequentially washed with water (10 mL), ethanol (10 mL), and CH_2Cl_2 (10 mL). The light-yellow powder of H_2**L** (0.83 g, 2.90 mmol, ~94.2% yield) was obtained after drying in a 120 °C oven for 30 minutes. ^{1}H NMR (400 MHz, DMSO-d_6): δ = 12.97 (s, 2 H), 8.23 (s, 2 H), 7.97 (s, 2 H), 7.68 (s, 8 H). The spectroscopic data are identical to those reported in the literature.[3] MS (ESI-TOF): calculated m/z for $[M + H]^+$ ($C_{18}H_{15}N_4$), 287.130; observed, 287.1295.

S1.2.3 Synthesis of {[Zn(L)] (H_2L)$_{1.5}$}$_n$ (ZnMOF). A mixture of 5 mg H_2**L** (17.5 μmol), 15 mg $Zn(NO_3)_2$ $6H_2O$ (50.4 μmol), 6 mL DEF and 0.9 mL water in an 8.5 mL vial was stirred with a magnetic bar at 1500 rpm at 80 °C for 9 h. The crude product was collected by centrifugation (2.0×10^4 rpm for 15 min), then dispersed in 5 mL DEF in an 8.5 mL vial. The vial was held in a 120 °C oven for 12 h to dissolve the trapped H_2**L**. After cooling to room temperature, the product was collected by centrifugation (2.0×10^4 rpm for 15 min) and washed with 10 mL water (5 times), and then redispersed in 10 mL water for further use.

S1.2.4 Synthesis of {[Zn(2-methylimidazol-1-ide)$_2$]}$_n$ (ZIF-8). ZIF-8 was prepared following the literature procedure.[4] 11.3 mL methanolic solution with 298 mg dissolved $Zn(NO_3)$ $6H_2O$ was added into 11.3 mL methanolic solution of 660 mg dissolved 2-methylimidazole in a 50 mL round-bottom flask. The mixed solution was further stirred at room temperature for 1 h. The product was centrifuged (2.0×10^4 rpm for 15 min), washed with 30 mL water (five times), and then redispersed in 30 mL water for further use.

S1.3. DOX Loading and Release Profiles

ZnMOF or ZIF-8 at a Zn concentration of 0.125 mM was co-incubated with 0.16 mM of DOX in water overnight and then the mixture was centrifuged. The supernatant was kept for UV–visible spectroscopy measurement to determine the concentration of remaining DOX. For release profiles, DOX@ZnMOF or DOX@ZIF-8 was freshly prepared and redispersed in H_2O, pH 5.5, 0.1×PBS, or PBS solutions in 1.5 mL Eppendorf (EP) tubes (3 replicates for each time point), respectively. The EP tubes were transferred to a 37 °C incubator. The supernatants were collected at 0, 2, 4, 8 and 24 h by centrifugation at 3,000 g and directly subjected to UV–visible spectroscopy for quantification.

S1.4. In Vitro Studies

Cell viability assay

CT26 cells were seeded in 96-well plates at a density of 5,000 cells/well and cultured overnight. ZnMOF was added at a Zn concentration of 0,3.7, 7.5, 15, 30, 60, 125, 250, and 500 µM and incubated for 24 hours (n = 4) followed by MTS assay. IC_{50} values of all treatment groups were fitted with the non-linear regression curves in GraphPad software.

CT26 cells were seeded in 96-well plates at a density of 5,000 or 1000 cells/well and cultured overnight. ZnMOF, ZIF-8, DOX@ZnMOF, DOX@ZIF-8 and DOX was added at a Zn concentration of 0, 25, 50, and 100 µM. For DOX, the concentrations were 0, 3.2, 6.5, and 13 µM. The cells were incubated for 24 or 72 hours (n = 4) followed by MTS assay. IC_{50} values of all treatment groups were fitted with the non-linear regression curves in Graghpad software.

Cellular uptake

CT26 cells were seeded in 6-well plates at a density of 2×10^5/well and incubated overnight. PBS, ZIF-8, or ZnMOF was added at a Zn concentration of 50 µM (n = 3). The cells were incubated at 37 °C for 4 or 24 hours. Then the medium was aspirated, and the cells were washed with PBS three times, trypsinized, and collected by centrifugation at 300 g for 3 minutes The cell pellets were digested with 1 mL of concentrated HNO_3 with 1% HF in 1.5 mL Eppendorf tubes for 48 hours. The Zn concentration was determined by ICP-MS after dilution.

Intracellular Zn^{2+} detection.

Intracellular Zn^{2+} detection was performed with a Zn^{2+} probe [N-(6-Methoxy-8-quinolyl)-p-toluenesulfonamide] (TSQ, AAT Bioquest, Inc.). CT26 cells were seeded into 24-well plates at a density of 5×10^4 cells per well (0.5 mL of 1640) and then incubated with PBS, ZIF-8, ZnMOF, or $Zn(NO_3)_2$ at a Zn concentration of 50 µM for 4 h. Afterwards, cells were either immediately stained or incubated in fresh cell culture medium for 24 h before staining. For TSQ staining, cells were washed with PBS and stained with 1 µL of TSQ DMSO solution (0.1 M) for 30 min. Next, the culture media were replaced and washed with fresh PBS and analyzed by flow cytometry.

To study mitochondrial targeting, free DOX, DOX@ZIF-8, or DOX@ZnMOF with 6.5 µM DOX or/and 50 µM ZnMOF (based on Zn) was added to CT26 cells and incubated for 6 hours. The cells were stained with Hoechst and mitotracker for 30 minutes. Afterwards, the cells were washed with PBS three times, exchanged with warm phenol-red-free RPMI-1640 medium, and mounted for confocal imaging immediately using a Leica Stellaris 8 microscope.

Western blot analysis

All antibodies used in western blot experiments were purchased from Cell Signaling Technology or ThermoFisher. All buffers, assays, and XCell SureLock™ Mini-Cell were from ThermoFisher. The mini trans-blot electrophoretic transfer cell was from Bio-Rad, and the FluorChem R system was from ProteinSimple. Cells were lysed with RIPA buffer containing protease and phosphatase inhibitor cocktail following the manufacturer's specifications. The proteins in the supernatant were collected by centrifugation at 14000 g, and the concentrations were measured

and normalized by BCA assay. The proteins were denatured and reduced by NuPAGE™ LDS sample buffer with 50 mM DTT, and then heated to 80 °C for 10 min. 10 to 20 μg of samples were loaded on NuPAGE™ 4 to 12%, Bis-Tris gel for electrophoresis on a XCell SureLock™ Mini-Cell (200V, 35-50 minutes), and electrotransferred to PVDF membrane (300 mA, 70 min) on a mini trans-blot electrophoretic transfer cell. The membrane was blocked by TBST with 5% non-fat dry milk at room temperature for 1 hour and incubated with primary antibody solution in TBST with 5% BSA at 4°C overnight (Phospho-histone H2A.X (Ser139) (20E3) rabbit mAb #9718, 1:2000; GPX4 rabbit mAb#52455, 1:1000. The membrane was washed with TBST and incubated with secondary antibody with HRP conjugate in TBST with 5% BSA at room temperature for 1 hour (anti-rabbit IgG, HRP-linked antibody #7074, 1:5000; anti-mouse IgG, HRP-linked antibody #7076, 1:5000). The membrane was again washed with TBST and Pierce™ ECL western blotting substrate was added. The chemiluminescence was then recorded on a FluorChem R system.

In vitro total ROS detection by DCFDA assay

Total ROS generation in vitro was measured using the 2',7'-dichlorodihydrofluorescein diacetate (DCFDA) assay kit (ThermoFisher, USA) following the vendor's protocol. Briefly, CT26 cells were plated in 35 mm tissue culture dishes overnight. PBS, ZnMOF, ZIF-8, DOX, DOX@ZnMOF or DOX@ZIF-8 at an equivalent Zn concentration of 50 μM was added to the wells and the cells were incubated for 24 hours. The cells were then washed with DPBS twice and exchanged with 10 μM DCFDA in prewarmed DMEM medium. The cells were incubated in a 37 °C incubator for 45 minutes and then washed with DPBS and stained with Hoechst and mounted for confocal imaging immediately using a Leica Stellaris 8 microscope.

In vitro lipid peroxidation.

10^5 CT26 cells were seeded in 6-well plates and incubated overnight. PBS or 10 μM ferrostatin 1 (Fer-1) was added. After incubation for 2 hours, PBS, ZnMOF, or ZIF-8 at an equivalent Zn concentration of 50 μM was added to PBS groups, while ZnMOF (50 μM based on Zn) was added to Fer-1 group. The cells were then incubated for 24 hours. After washing with PBS three times, the cells were stained with Liperfluo (Cayman Chemical) for 40 min. The cells were washed with PBS and immediately harvested for flow cytometry.

In vitro DNA damage by γ-H_2AX assay

γ-H_2AX, a protein that is phosphorylated after oxidation by hydroxyl radicals to induce DNA damage repair, has been used as a sensitive biomarker for probing DNA double strand breaks. CT26 cells were plated in 24-well plates overnight and incubated with PBS, ZnMOF, ZIF-8, DOX, DOX@ZnMOF, or DOX@ZIF-8 at an equivalent Zn concentration of 50 μM. After incubation for 24 additional hours, the cells were washed with PBS, collected following fixation with 4% paraformaldehyde and permeabilization with the permeabilize buffer (PBS + 1%FBS + 0.3% Triton-X), and resuspend in diluted primary antibody Phospho-histone H2A.X (Ser139) in flow buffer (DPBS with 0.5% BSA) at 1:200 dilution and cultured for 1 h at room temperature. The cells were then washed with flow buffer twice and resuspended in 100 μl of diluted

fluorophore-conjugated secondary antibody 1:2000 for 30 min at room temperature in the dark. After washing, the cells were resuspended in flow buffer and analyzed on a flow cytometer.

Apoptosis assay

Apoptosis was evaluated on CT26 cells by flow cytometry. CT26 cells were seeded at a density of 5×10^4 cells/well and cultured overnight on 24-well plates. The cells were treated with PBS, ZnMOF, ZIF-8, DOX, DOX@ZnMOF, or DOX@ZIF-8 at an equivalent Zn concentration of 50 μM,and further incubated for 48 hours. The cells were then scraped off and stained with Alexa Fluor 488 Annexin V/dead cell apoptosis kit (ThermoFisher, USA) following the vendor's protocol for flow cytometry analysis.

Cell cycle analysis

For cell cycle analysis, CT26 cells were seeded in 24-well plates at a density of 5×10^4 cells/well and cultured overnight. The cells were then treated with PBS, ZnMOF, ZIF-8, DOX, DOX@ZnMOF, or DOX@ZIF-8 at an equivalent Zn concentration of 50 μM. After incubation for another 24 h, the cells were collected by adding 200 μL of 0.05% trypsin-containing EDTA to each well and centrifugation (300 g) for 5 min. The cells were fixed using 3 mL ice-cooled 70% ethanol overnight and then centrifuged at 300 g for 5 min. The precipitate was treated with 450 μL of Hoechst staining solution. After overnight incubation, flow cytometry analysis was performed. All experiments were performed in triplicate.

S1.5 In Vivo Study

Antitumor Efficacy

For the evaluation of antitumor efficacy of DOX@ZnMOF, syngeneic models were established by inoculating 2×10^6 CT26 or MC38 cells onto the right flank subcutaneous tissues of Balb/c or C57 mice, respectively, on day 0. When the tumors reached ~100 mm^3 in volume, PBS, ZnMOF, ZIF-8, DOX, DOX@ZnMOF, or DOX@ZIF-8 at a Zn dose of 3 μmol and/or DOX dose of 0.39 μmol was injected intratumorally on days 7 and 8. The tumor sizes were measured with a caliper every day where tumor volume equals ($width^2 \times$ length)/2. Body weights of the mice were also monitored every day. The mice were sacrificed when the PBS group reach the endpoint. Tumors and major organs were sectioned for hematoxylin-eosin staining (H&E), immunofluorescent TUNEL analysis, as well as immunohistochemistry staining for phospho-histone H_2A.X and GPX4. The tumor growth inhibition index (TGI) was defined as:

$$TGI = (1 - \frac{Te-Ts}{Ce-Cs}) \times 100\%$$

where Te, Ts, Ce, and Cs represent average tumor volumes of treated mice at endpoint, treated mice at starting-point, control mice at endpoint and control mice at starting-point, respectively.

Serum Biochemistry (AST, ALT, BUN)

A separate CT26 tumor-bearing mouse model was used to evaluate potential hepatic and renal toxicity after treatment. At 48 h post-treatment, blood samples were collected via retro-orbital bleeding and centrifuged at 5,000 rpm for 10 min to obtain serum. The collected serum was transferred to RNase-free tubes and analyzed for aspartate aminotransferase (AST), alanine aminotransferase (ALT), and blood urea nitrogen (BUN) levels using commercial assay kits (Alfa Wassermann) according to the manufacturer's instructions. Measurements were performed using a Vet Axcel Chemistry Analyzer. All samples were analyzed within the same batch to minimize inter-assay variability. The results, expressed as mean ± SD, were used to assess possible hepatic or renal toxicity following various treatments.

Hemolysis Assay

To assess hemocompatibility, a hemolysis assay was performed using fresh anticoagulated whole blood collected from mice. Blood samples were centrifuged at 3,000 rpm for 10 min to remove plasma, and red blood cells (RBCs) were washed three times with PBS and resuspended to prepare an RBC suspension. Various concentrations of ZnMOF or DOX@ZnMOF were incubated with the RBC suspension (final volume: 200 µL) at 37 °C for 3–4 h. After incubation, the samples were centrifuged at 3,000 rpm for 5 min, and the absorbance of the supernatant was measured at 540 nm using a microplate reader.

PBS and 1% Triton X-100 served as negative (0% hemolysis) and positive (100% hemolysis) controls, respectively. The hemolysis percentage was calculated using the standard formula,

$$Hemolysis\ (\%)\ = \frac{A_{sample} - A_{PBS}}{A_{Triton} - A_{PBS}} \times\ 100\%$$

All measurements were performed in triplicate. A hemolysis rate below **5%** was considered indicative of excellent hemocompatibility.

S1.6 Statistical analysis

All statistical analysis was performed on Origin Lab software by pair-sample t-test or One-way Repeated Measures ANOVA method. The p values were defined as * p<0.05, ** p<0.01, *** p<0.001, **** p<0.0001 in all figures.

S1.7 Ethical statement

This research complies with all relevant ethical regulations. All work performed on animals was in accordance with and approved by the Institutional Animal Care and Use Committee at the University of Chicago. The approved number is 72408. Animals were housed in 12 light/12 dark cycle, 65-75°F (~18-23°C), and 40-60% humidity condition. Animals were euthanized when the

tumor reached 20 mm in any dimension or when they became moribund with severe weight loss or unhealing ulceration. This limit was not exceeded at any point.

S2 Supporting Figures and Tables

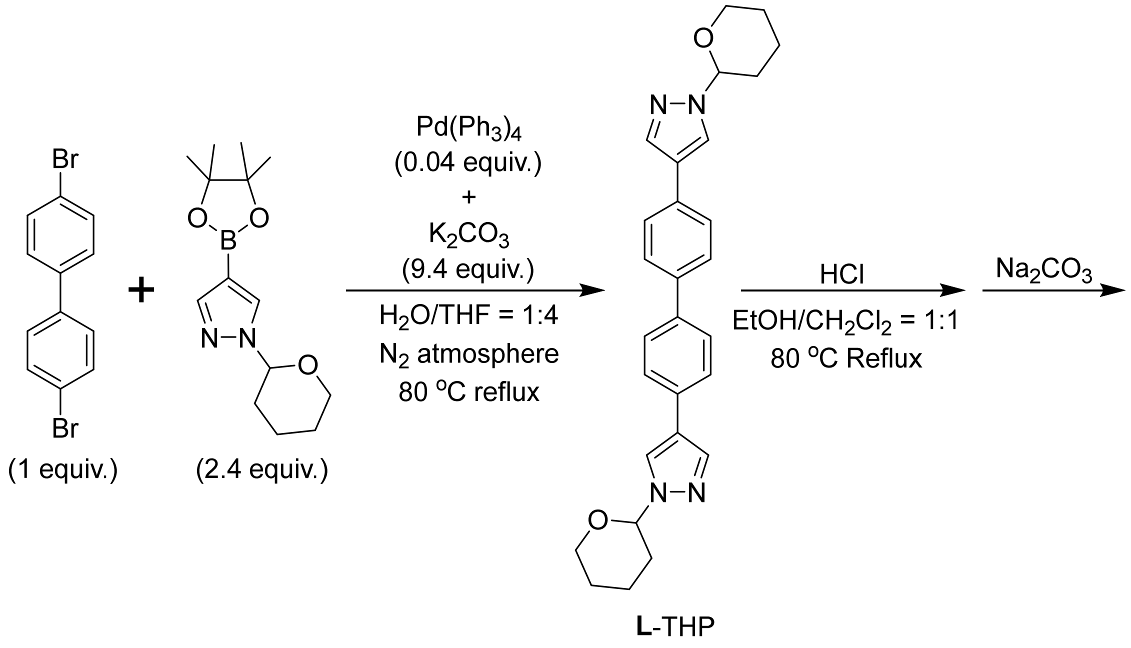

Figure S1. Schematic illustration of the synthetic procedure for H_2**L**.

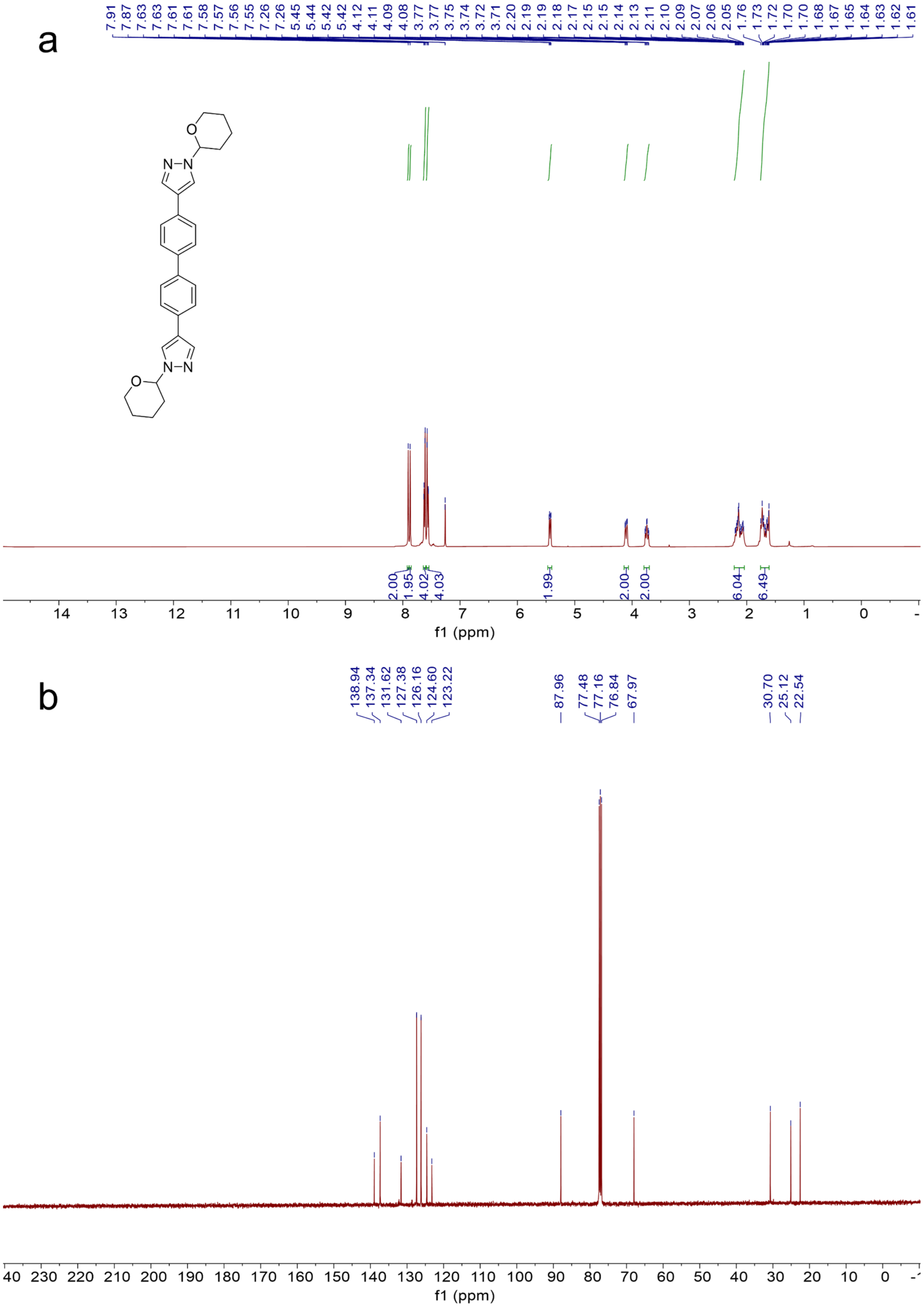

Figure S2. (a) ^{1}H and (b) $^{13}C\{^{1}H\}$ NMR spectra of **L**-THP.

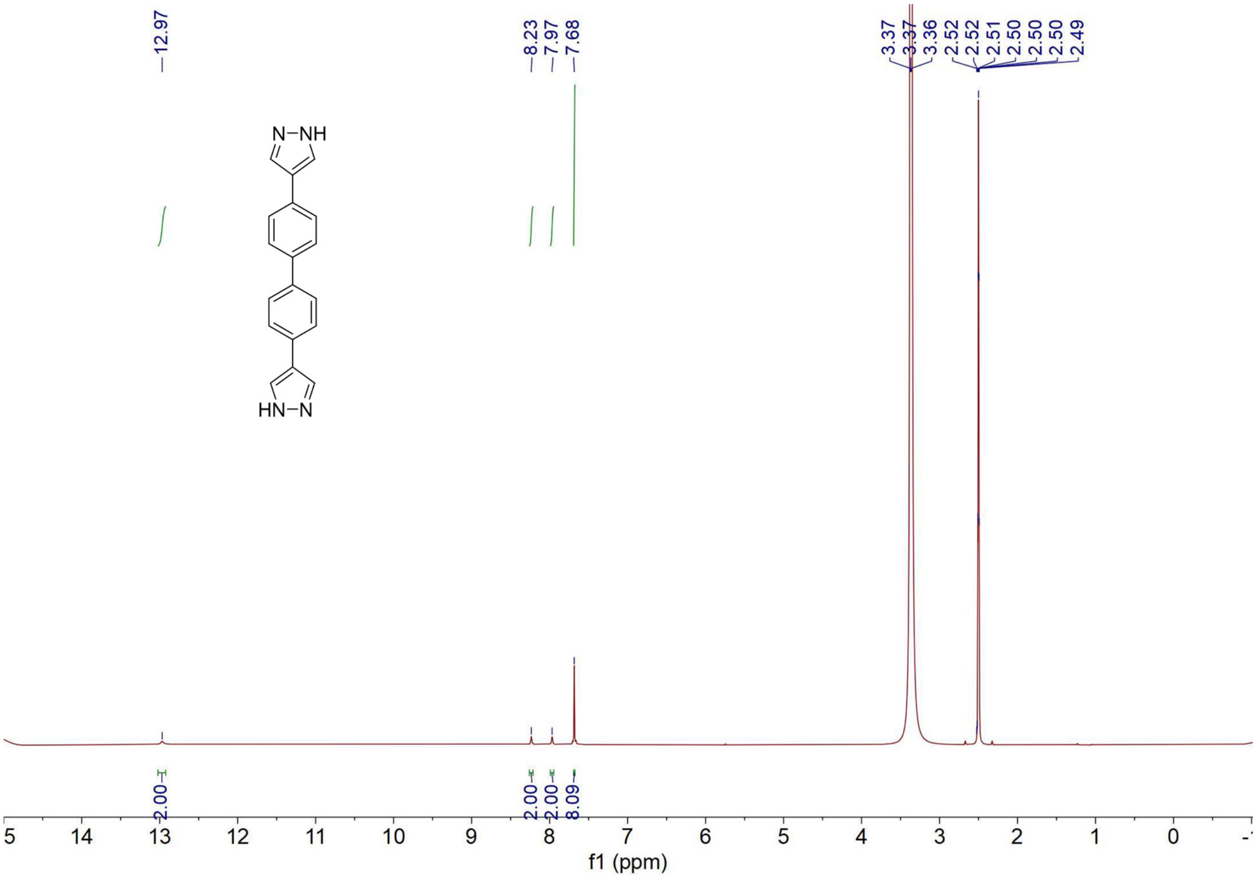

Figure S3. ^{1}H NMR spectrum of H_2**L**.

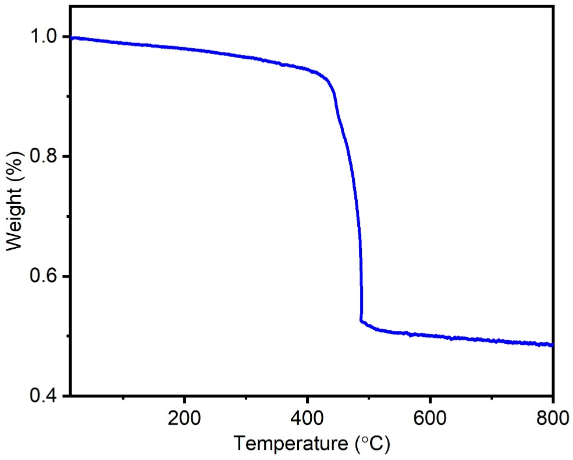

Figure S4. TGA curve of ZnMOF.

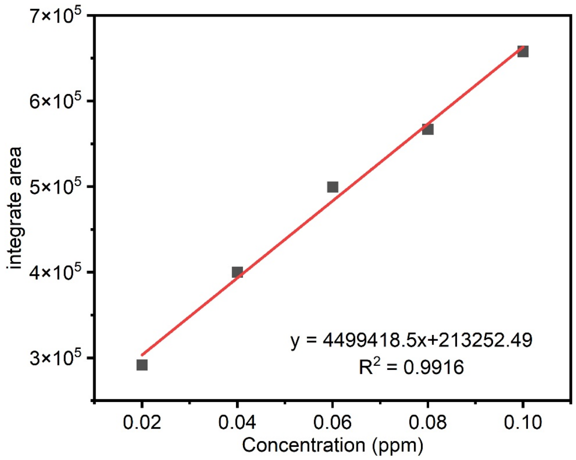

Figure S5. Calibration curve of H_2**L** by LC-MS. A mixed solvent of H_2O/TFA = 2:1 (v/v) was used for dissolving H_2**L** and digesting ZnMOF.

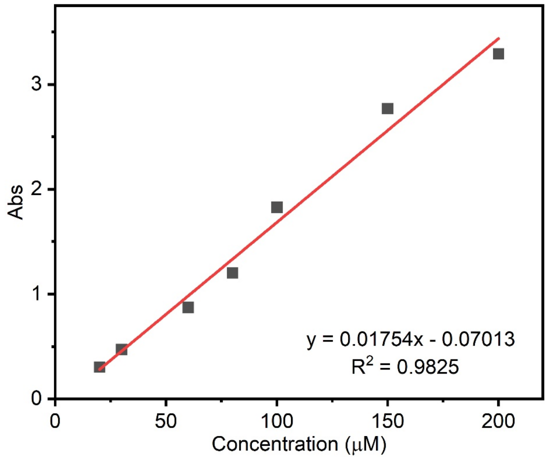

Figure S6. Calibration curve of H_2**L** at 292 nm obtained by UV-vis spectroscopy. A mixed solvent system of H_2O/TFA/DMSO = 1:2:3 (v/v/v) was used for dissolving H_2**L** and digesting ZnMOF.

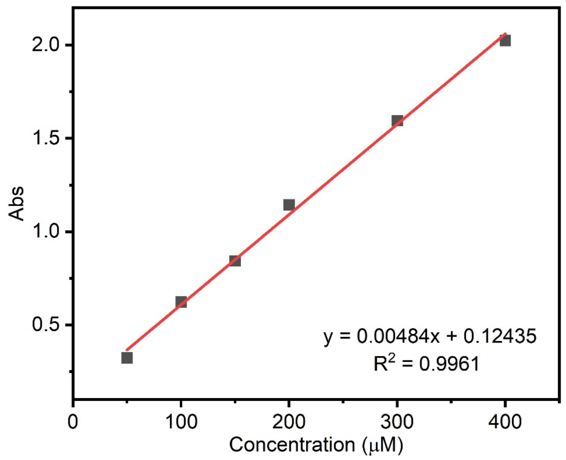

Figure S7. Calibration curve of 2-methylimidazole at 209 nm obtained by UV-vis spectroscopy. A 1.4 wt% aqueous ammonium hydroxide solution was used for dissolving 2-methylimidazole and digesting ZIF-8.

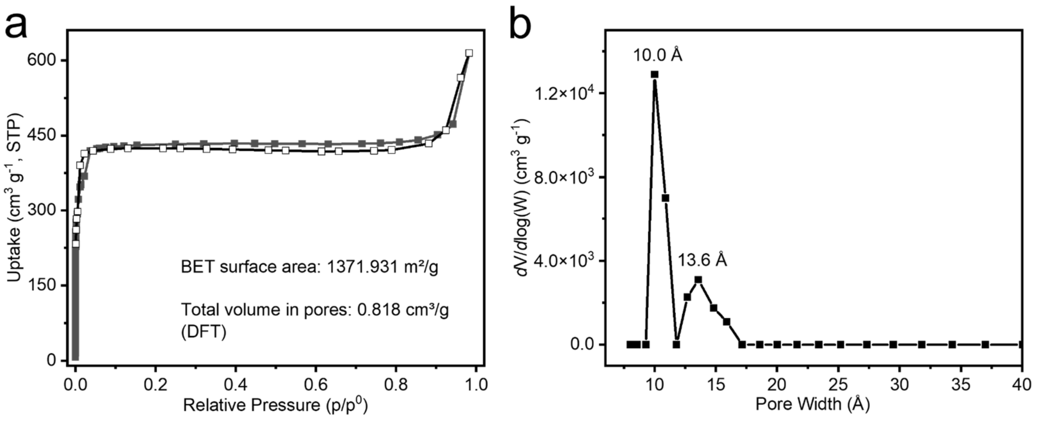

Figure S8. (a) Nitrogen sorption isotherms of ZIF-8 at 77 K. (b) Pore size distribution calculated by the density functional theory (DFT) method.

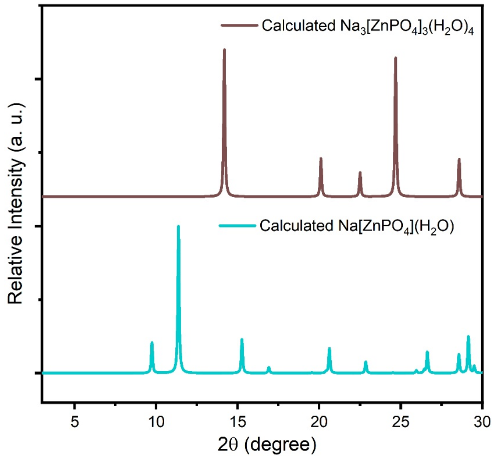

Figure S9. Calculated PXRD patterns of $Na_3[ZnPO_4]_3(H_2O)_4$ (ICSD63495) and $Na[ZnPO_4](H_2O)$ (ICSD81368).

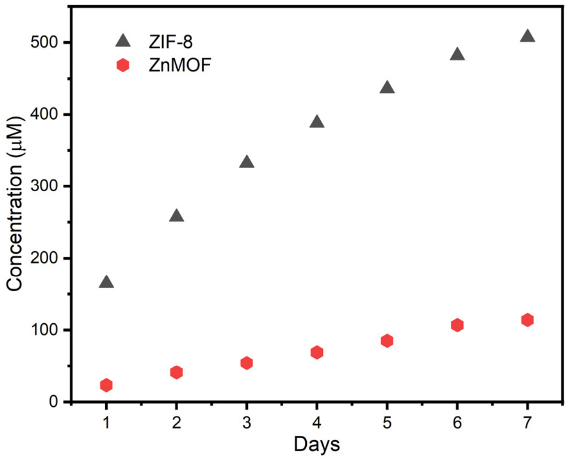

Figure S10. Ligand release from ZIF-8 and Zn-MOF after incubation in PBS (0.34 mM Zn) for one week, as determined by UV-vis absorption spectroscopy.

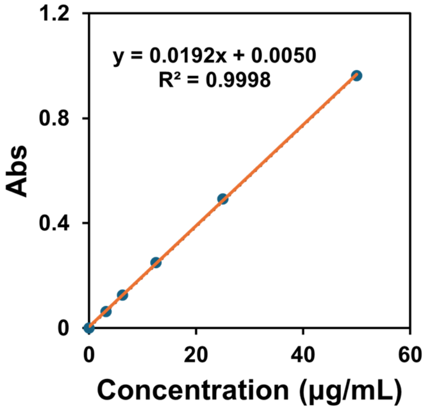

Figure S11. Calibration curve of DOX at 480 nm obtained by UV–Vis spectroscopy.

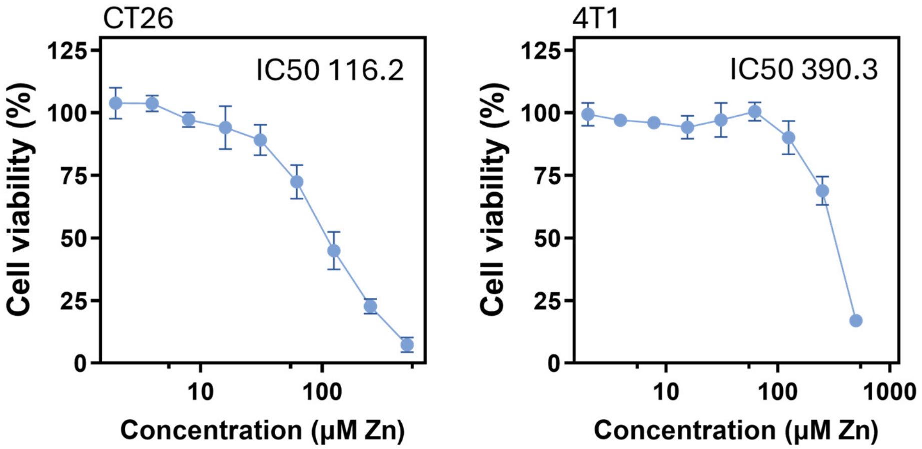

Figure S12. Cell viability curves (mean ± SD %) of CT26 or 4T1 cells after treatment with ZnMOF, n=3. The IC_{50} values of ZnMOF in CT26 and 4T1 cells were 116.2 and 390.3 μM, respectively.

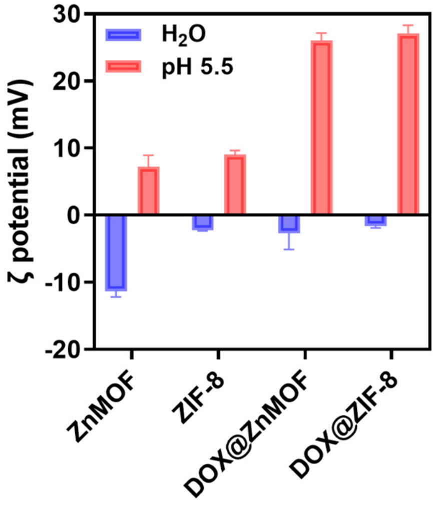

Figure S13. Zeta potentials of ZIF-8, ZnMOF, DOX@ZIF-8, and DOX@ZnMOF in water and at pH 5.5.

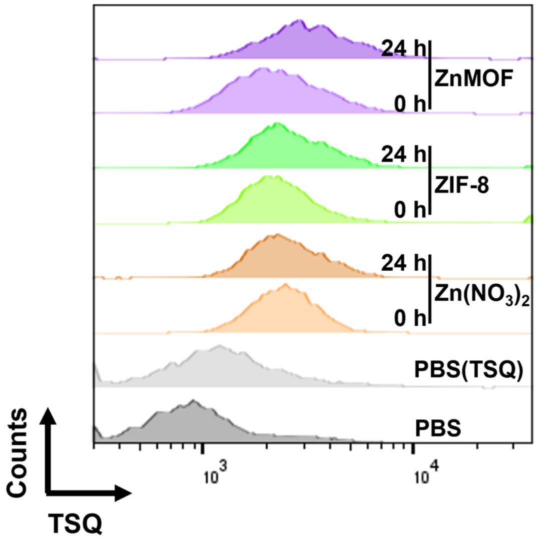

Figure S14. Flow cytometry histograms showing TSQ fluorescence after 0-h or 24-h incubation with fresh cell culture medium. ZnMOF-treated cells displayed the strongest TSQ signal, especially after 24 h incubation, indicating sustained Zn^{2+} release and intracellular accumulation.

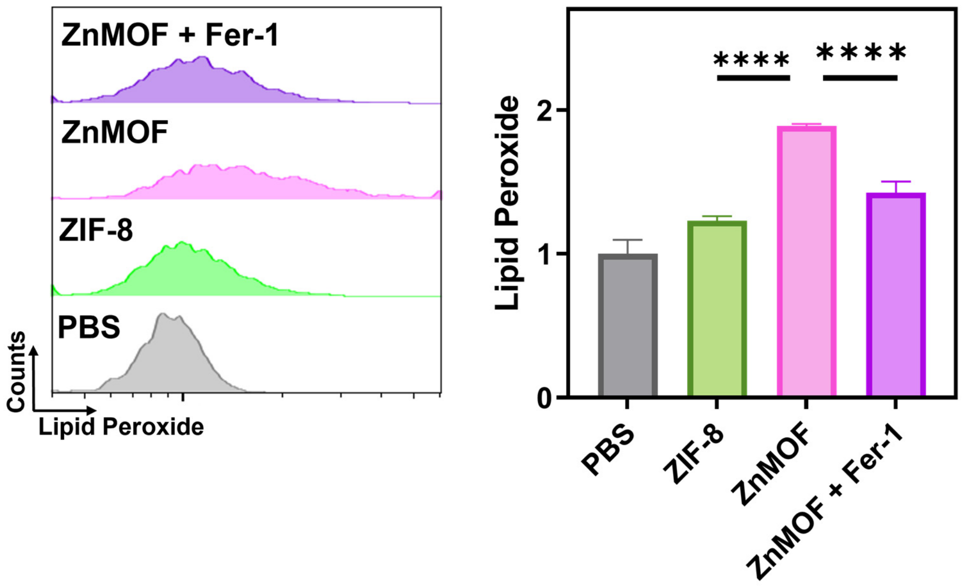

Figure S15. Flow cytometry histograms (left) and quantification (right) of lipid peroxide levels in CT26 cells after different treatments (n=3).

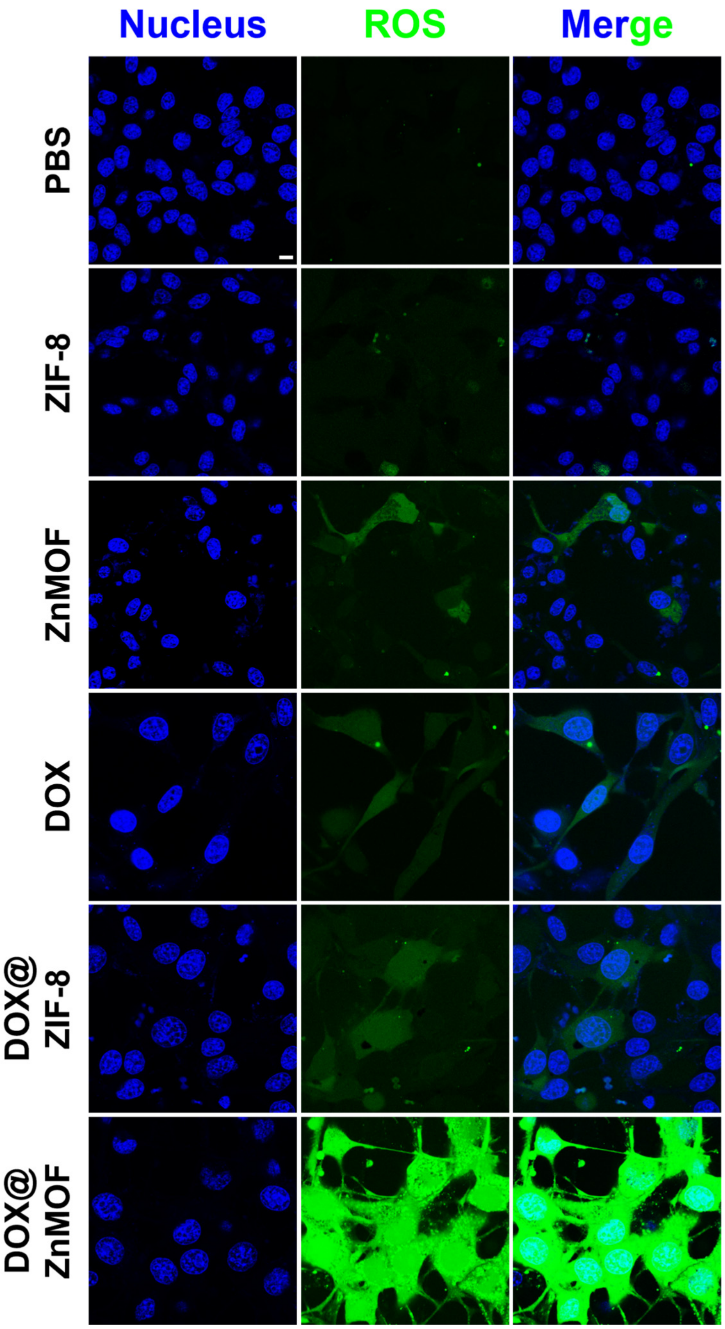

Figure S16. CLSM images of total ROS in CT26 cells after different treatments (Hoechst, blue; ROS, green; scale bar = 10 μm).

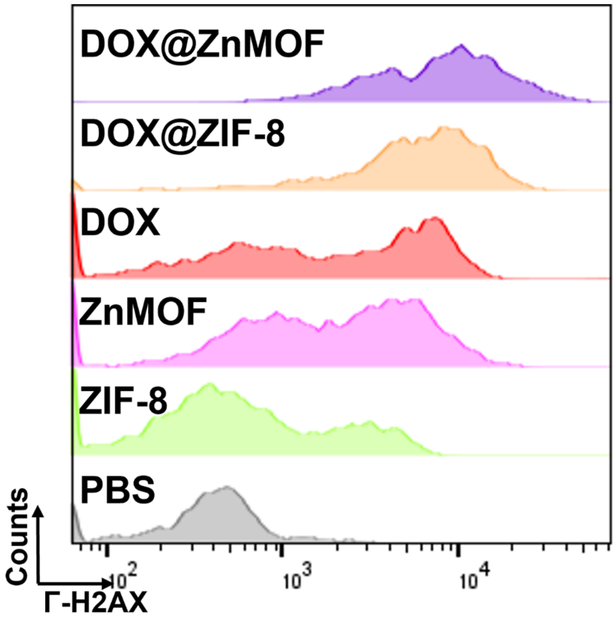

Figure S17. Flow cytometry histograms of γ-H2AX in CT26 cells (n=3).

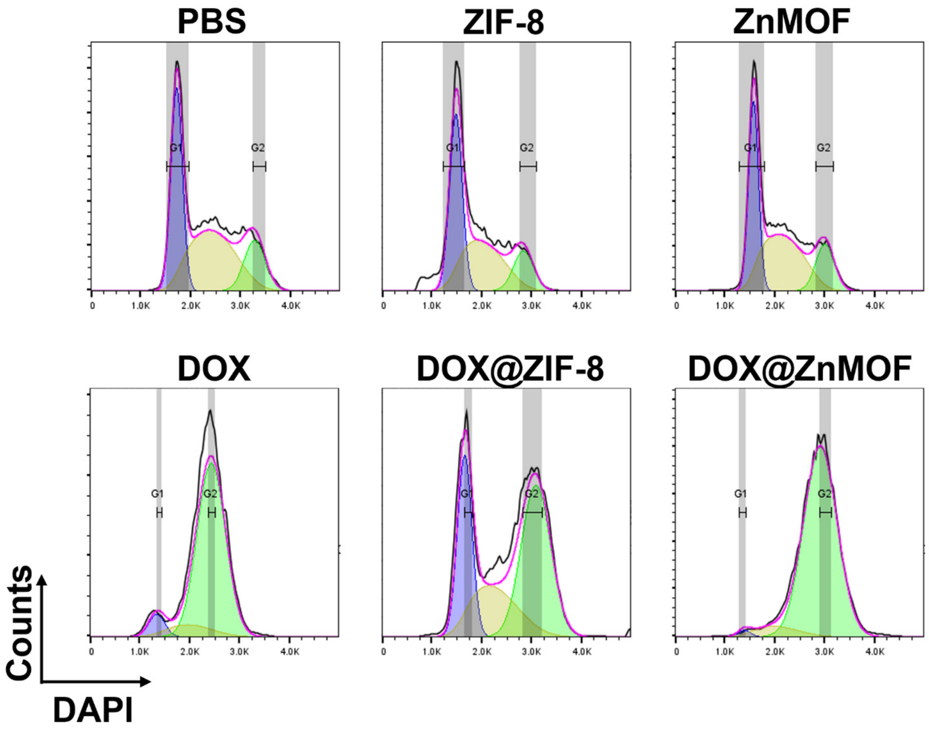

Figure S18. Cell cycle assay of CT26 cells after treatment with PBS, ZIF-8, ZnMOF, DOX, DOX@ZIF-8, or DOX@ZnMOF (n=3).

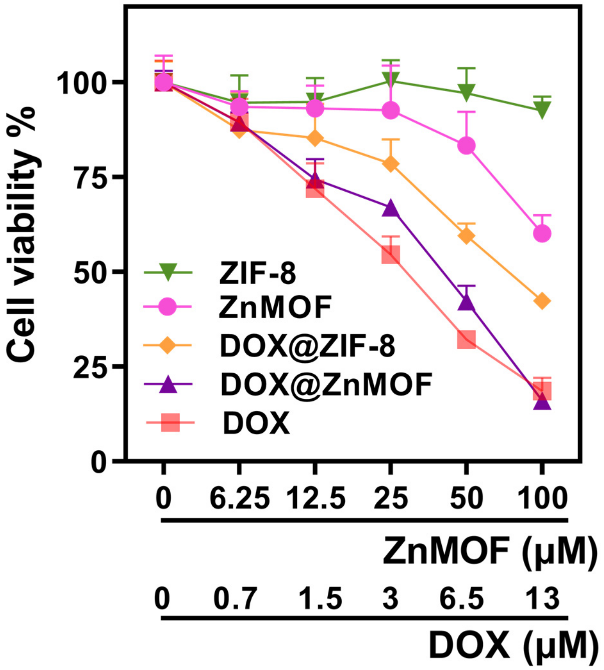

Figure S19. Cell viability curves (mean ± SD %) of CT26 cells after treatment with PBS, ZIF-8, ZnMOF, DOX, DOX@ZIF-8, or DOX@ZnMOF (n=3).

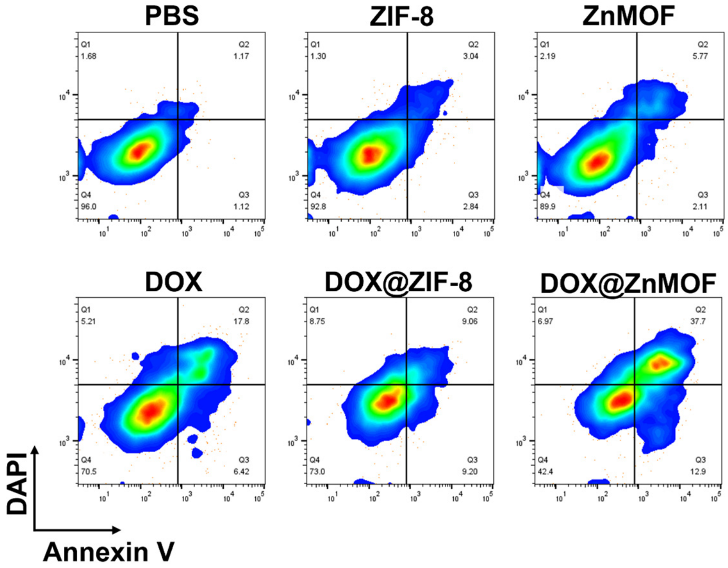

Figure S20. Apoptosis assay of CT26 cells after treatment with PBS, ZIF-8, ZnMOF, DOX, DOX@ZIF-8, or DOX@ZnMOF (n=3).

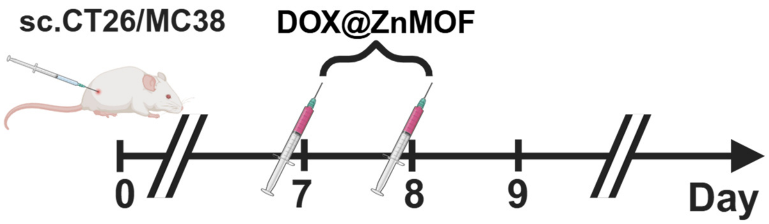

Figure S21. Tumor inoculation and treatment schedules. All treatments were intratumorally injected on days 7 and 8 post tumor inoculation. Created with BioRender.

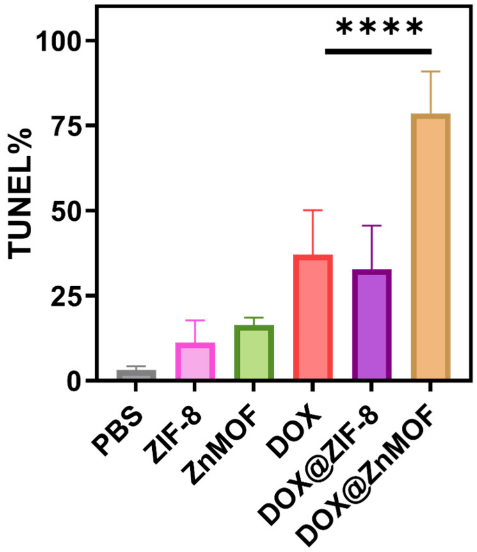

Figure S22. Quantification of TUNEL-positive apoptotic cells across different treatment groups, n=5. Statistical significance: ns, not significant; *$p<0.05$, **$p<0.01$, ***$p<0.001$, ****$p<0.0001$.

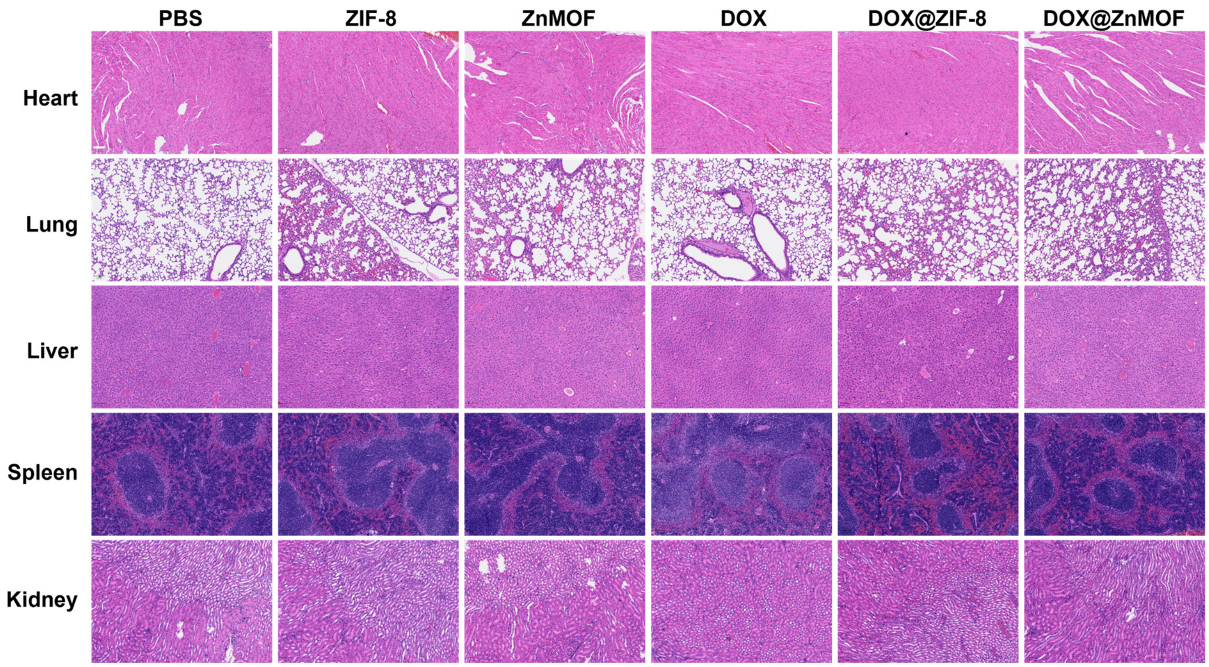

Figure S23. H&E staining of heart, lung, liver, spleen, kidney from CT26 tumor-bearing BALB/c mice in different treatment groups (Scale bar = 100 μm).

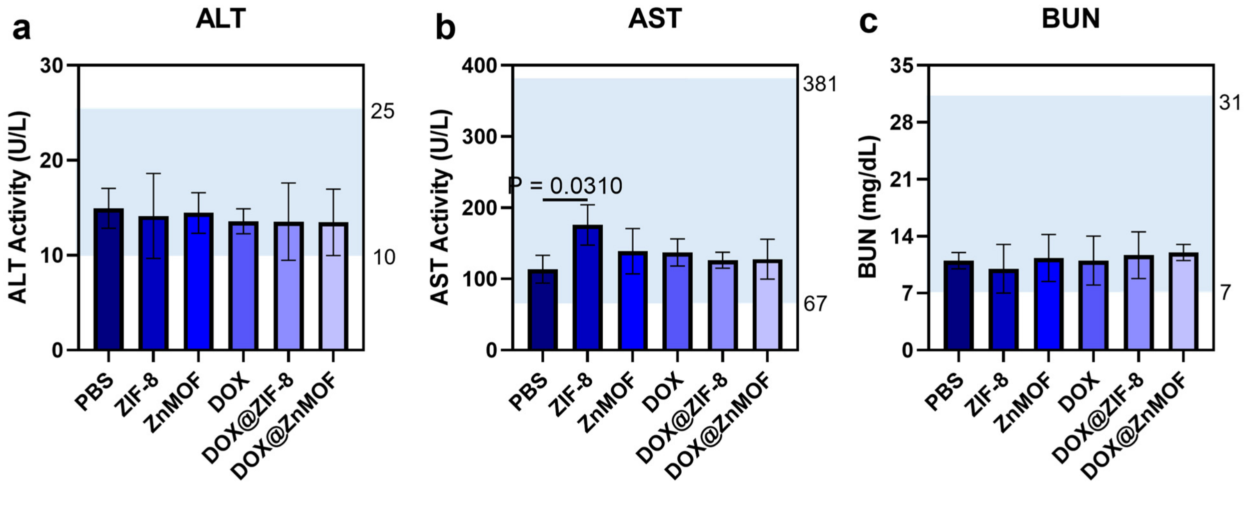

Figure S24. Serum biochemistry analysis of mice after different treatments. Quantification of ALT (a), AST (b), and BUN (c) levels in serum collected 48 h post-treatment. The normal physiological range is indicated on the right axis. Data are presented as mean ± SD (n = 3).

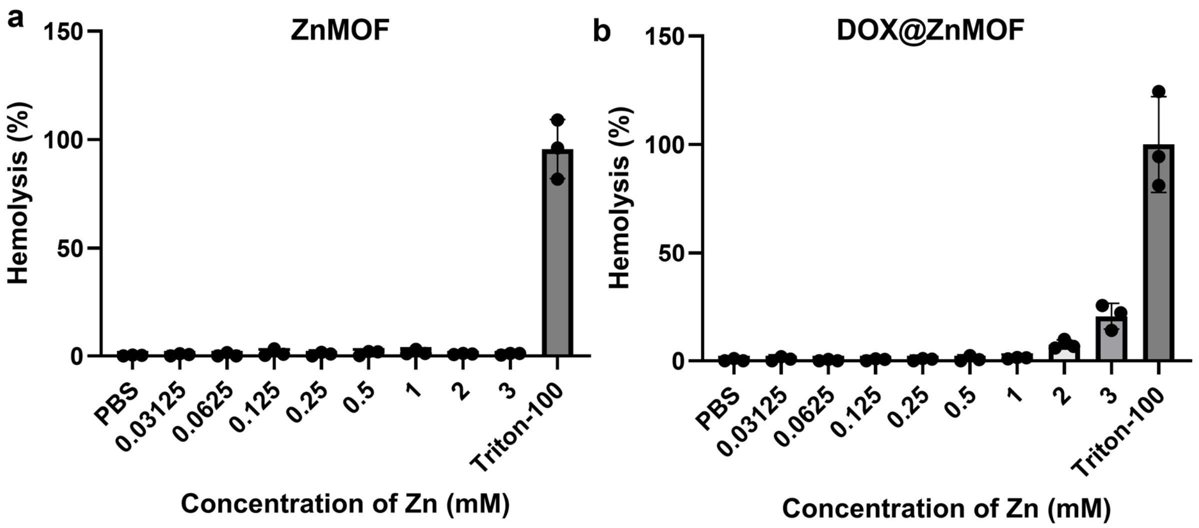

Figure S25. Hemolysis assay of ZnMOF and DOX@ZnMOF. Hemolysis percentages of mouse red blood cells after incubation with various concentrations of ZnMOF (a) or DOX@ZnMOF (b) at 37 °C for 3–4 h. PBS and 1% Triton X-100 served as negative (0%) and positive (100%) controls, respectively. Thus, ZnMOF shows excellent hemocompatibility at up to 3 mM Zn. At therapeutically relevant doses (<1 mM Zn), DOX@ZnMOF also showed negligible hemolysis.

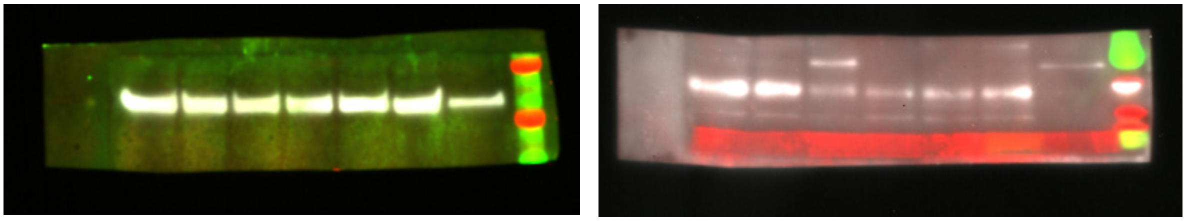

Figure S26. Uncropped blot for Figure 4c. Expression of GPX4 (right) with β-actin as the reference (left).

Table S1 Fractional atomic coordinates of ZnMOF from Pawley refinements.

Space Group: *Pc*<br>a = 6.1110 Å, b = 30.4782 Å, c= 20.6927 Å, α = γ = 90°, β = 110.0354°, V = 3620.81903 Å^3				
Element	Atom	x	y	z
C	C1	-0.38187	0.60619	-0.62998
C	C2	-0.56679	0.57745	-0.63269
C	C3	-0.59984	0.53673	-0.66826
C	C4	-0.4492	0.52231	-0.70285
C	C5	-0.26339	0.55094	-0.69988
C	C6	-0.23104	0.59184	-0.66457
C	C7	-0.48653	0.47812	-0.74155
C	C8	-0.29842	0.45141	-0.74291

C	C9	-0.33394	0.41048	-0.77818
C	C10	-0.55945	0.39415	-0.8143
C	C11	-0.7473	0.42097	-0.81319
C	C12	-0.71185	0.46173	-0.7777
C	C13	-0.34734	0.65033	-0.5915
C	C14	-0.59912	0.34994	-0.85264
C	C15	-0.15796	0.67936	-0.57449
N	N16	-0.18831	0.71256	-0.53435
N	N17	-0.38939	0.70776	-0.52706
C	C18	-0.48919	0.67018	-0.55921
C	C19	-0.80816	0.32776	-0.8849
N	N20	-0.77063	0.29092	-0.91656
N	N21	-0.55235	0.2891	-0.91008
C	C22	-0.44027	0.32318	-0.86964
Zn	Zn23	-0.4749	0.74888	-0.47196
Zn	Zn24	0.0251	0.75113	-0.47196
C	C25	-0.05945	-0.10585	-0.3143
C	C26	-0.2473	-0.07903	-0.31319
C	C27	-0.21185	-0.03827	-0.2777
C	C28	0.01347	-0.02188	-0.24155
C	C29	0.20158	-0.04859	-0.24291
C	C30	0.16606	-0.08952	-0.27818
C	C31	0.0508	0.02231	-0.20285
C	C32	0.23661	0.05094	-0.19988
C	C33	0.26896	0.09184	-0.16457
C	C34	0.11813	0.10619	-0.12998
C	C35	-0.06679	0.07745	-0.13269
C	C36	-0.09984	0.03673	-0.16826
C	C37	-0.09912	-0.15006	-0.35264
C	C38	0.15266	0.15033	-0.0915
C	C39	0.05973	-0.17682	-0.36964
N	N40	-0.05235	-0.2109	-0.41008
N	N41	-0.27063	-0.20908	-0.41656
C	C42	-0.30816	-0.17224	-0.3849
C	C43	0.01081	0.17018	-0.05921
N	N44	0.11061	0.20776	-0.02706
N	N45	0.31169	0.21256	-0.03435
C	C46	0.34204	0.17936	-0.07449
H	H47	-0.68649	0.5861	-0.60654
H	H48	-0.74247	0.51598	-0.66744
H	H49	-0.14445	0.54215	-0.72628
H	H50	-0.08787	0.61251	-0.6651
H	H51	-0.12192	0.46173	-0.71523
H	H52	-0.18308	0.3913	-0.7763

H	H53	-0.92405	0.41074	-0.84058
H	H54	-0.86287	0.48097	-0.77982
H	H55	-0.00355	0.67511	-0.58732
H	H56	-0.64579	0.65637	-0.55532
H	H57	-0.97761	0.33974	-0.88994
H	H58	-0.25826	0.32964	-0.85598
H	H59	-0.42405	-0.08926	-0.34058
H	H60	-0.36287	-0.01903	-0.27982
H	H61	0.37808	-0.03827	-0.21523
H	H62	0.31692	-0.1087	-0.2763
H	H63	0.35555	0.04215	-0.22628
H	H64	0.41213	0.11251	-0.1651
H	H65	-0.18649	0.0861	-0.10654
H	H66	-0.24247	0.01598	-0.16744
H	H67	0.24174	-0.17036	-0.35598
H	H68	-0.47761	-0.16026	-0.38994
H	H69	-0.14579	0.15637	-0.05532
H	H70	0.49645	0.17511	-0.08732

Table S2. TGI values of CT26-bearing BABL/c mice on day 12.

**Treatment**	**TGI**
ZIF-8	0.240
ZnMOF	0.382
DOX	0.695
DOX@ZIF-8	0.476
DOX@ZnMOF	0.910

Table S3. TGI values of MC38-bearing C57 mice on day 14.

**Treatment**	**TGI**
ZIF-8	0.409
ZnMOF	0.503
DOX	0.762

DOX@ZIF-8	0.562
DOX@ZnMOF	0.926

Table S4. Statistical analysis of treatment comparisons in CT26-bearing BALB/c mice on day 12.

CT26	Summary	Individual P Value
PBS vs. DOX	****	<0.0001
PBS vs. ZIF-8	ns	0.1206
PBS vs. ZnMOF	**	0.0062
PBS vs. DOX@ZIF-8	****	<0.0001
PBS vs. DOX@ZnMOF	****	<0.0001
DOX vs. ZIF-8	**	0.0065
DOX vs. ZnMOF	*	0.0365
DOX vs. DOX@ZIF-8	ns	0.3473
DOX vs. DOX@ZnMOF	*	0.0136
ZIF-8 vs. ZnMOF	ns	0.1746
ZIF-8 vs. DOX@ZIF-8	*	0.0129
ZIF-8 vs. DOX@ZnMOF	**	0.0022
ZnMOF vs. DOX@ZIF-8	ns	0.0891
ZnMOF vs. DOX@ZnMOF	**	0.0051
DOX@ZIF-8 vs. DOX@ZnMOF	**	0.0022

The p-values represent the significance of differences between various treatment groups, determined using an appropriate statistical test. *p<0.05, **p<0.01, ***p<0.001, ****p<0.0001; ns = not significant.

Table S5. Statistical analysis of treatment comparisons in MC38-bearing C57 mice on day 14.

MC38	Summary	Individual P Value
PBS vs. DOX	****	<0.0001

PBS vs. ZIF-8	****	<0.0001
PBS vs. ZnMOF	****	<0.0001
PBS vs. DOX@ZIF-8	****	<0.0001
PBS vs. DOX@ZnMOF	****	<0.0001
DOX vs. ZIF-8	****	<0.0001
DOX vs. ZnMOF	****	<0.0001
DOX vs. DOX@ZIF-8	**	0.0036
DOX vs. DOX@ZnMOF	*	0.0413
ZIF-8 vs. ZnMOF	ns	0.0593
ZIF-8 vs. DOX@ZIF-8	****	<0.0001
ZIF-8 vs. DOX@ZnMOF	****	<0.0001
ZnMOF vs. DOX@ZIF-8	*	0.0191
ZnMOF vs. DOX@ZnMOF	****	<0.0001
DOX@ZIF-8 vs. DOX@ZnMOF	****	<0.0001

The p-values represent the significance of differences between various treatment groups, determined using an appropriate statistical test. *p<0.05, **p<0.01, ***p<0.001, ****p<0.0001; ns = not significant.

References

[1] P. Bankhead, M. B. Loughrey, J. A. Fernández, Y. Dombrowski, D. G. McArt, P. D. Dunne, S. McQuaid, R. T. Gray, L. J. Murray, H. G. Coleman, J. A. James, M. Salto-Tellez, P. W. Hamilton, M. B. Loughrey, J. A. Fernández, Y. Dombrowski, D. G. McArt, P. D. Dunne, S. McQuaid, R. T. Gray, L. J. Murray, H. G. Coleman, J. A. James, M. Salto-Tellez, P. W. Hamilton, *Sci. Rep.* **2017**, *7*, 16878.

[2] S. Galli, N. Masciocchi, V. Colombo, A. Maspero, G. Palmisano, F. J. López-Garzón, M. Domingo-García, I. Fernández-Morales, E. Barea, J. A. R. Navarro, *Chem. Mater.* **2010**, *22*, 1664-1672.

[3] L. J. Kershaw Cook, R. Kearsey, J. V. Lamb, E. J. Pace, J. A. Gould, *Tetrahedron Lett.* **2016**, *57*, 895-898.

[4] K. Kida, M. Okita, K. Fujita, S. Tanaka, Y. Miyake, *CrystEngComm* **2013**, *15*, 1794-1801.
